# Clinical application of liquid biopsy in cancer patients

**DOI:** 10.1186/s12885-022-09525-0

**Published:** 2022-04-15

**Authors:** Chieh-Min Chang, Kuei-Ching Lin, Nien-En Hsiao, Wei-An Hong, Chia-Yu Lin, Ta-Chih Liu, Ya-Sian Chang, Jan-Gowth Chang

**Affiliations:** 1 Graduate Institute of Biomedical Sciences, China Medical University, Taichung, Taiwan; 2 Epigenome Research Center, China Medical University Hospital, Taichung, Taiwan; 3 Center for Precision Medicine, China Medical University Hospital, 2 Yuh-Der Road, Taichung, 404 Taiwan; 4 Department of Laboratory Medicine, China Medical University Hospital, Taichung, Taiwan; 5 Department of Hematology-Oncology, Chang Bing Show Chwan Memorial Hospital, 6 Lugong Road, Changhua, 505 Taiwan; 6 School of Medicine, China Medical University, Taichung, Taiwan; 7 Department of Bioinformatics and Medical Engineering, Asia University, Taichung, Taiwan

**Keywords:** Liquid biopsy, Clonal hematopoiesis, Somatic mutation, Germline mutation, Pathogenic/likely pathogenic variant

## Abstract

**Background:**

This study was to determine the prevalence and clinical significance of clonal hematopoiesis (CH)-related variants, and somatic and germline mutations in cancer patients and healthy individuals.

**Methods:**

We performed next-generation sequencing of 275 cancer-related genes be-tween plasma and white blood cells in 92 cancer patients and 47 controls without cancer. Blood samples were recruited from May 2017 to July 2021, and blood cancer patients were excluded. For all statistical analysis in this study, *p* < 0.05 was considered statistically significant.

**Results:**

Overall, 38.04% of patients and 46.81% of controls harbored at least one CH-related mutation in plasma cell-free DNA. Based on our results, older cancer patients exhibited a CH phenomenon more frequently than younger patients (*p* = 0.0024). A total of 39 somatic pathogenic (P)/likely pathogenic (LP) mutations were identified in 17 genes in 21 of 92 patients. We found that the presence of P/LP variants in cancer-related gene predicted shorter overall survival (OS) (*p* = 0.001). Multivariate analysis adjusted for CH-related mutations, germline mutations, and tumor stage, also indicated that somatic mutations correlated significantly with OS (*p* = 0.022). Moreover, the frequency of a germline P/LP variant was that of seven of 92 individuals in the cancer group and one of 42 individuals in the control group.

**Conclusions:**

We characterized the CH-related variants, and somatic and germline mutations in cancer patients and healthy individuals, and the results have important clinical significance.

**Supplementary Information:**

The online version contains supplementary material available at 10.1186/s12885-022-09525-0.

## Background

Liquid biopsy is a comprehensive and real-time analysis of tumor cells or tumor cell products released into the blood or other bodily fluids by all metastatic or primary tumor sites. Clinical application of liquid biopsy includes early detection of cancer or tumor recurrence, monitoring of cancer therapies, and determining therapeutic targets and resistance mechanisms to adapt therapy to the specific needs of an individual patient [1]. For example, liquid biopsy analysis has been demonstrated to allow detection of breast cancer 5 months earlier than traditional clinical examination [2]. Several immunotherapeutic drugs have been tested in clinical trials that use circulating tumor cells (CTCs) and circulating tumor-derived DNA (ctDNA) as biomarkers (www.clinicaltrials.gov). In addition to CTCs and ctDNA, members of the liquid biopsy marker family include extracellular vesicles [3], microRNAs [4], and tumor-educated platelets [5].

The presence of cell-free DNA (cfDNA) in human blood was first described by Mandel and Metais in 1948 [6]. For cancer patients, cfDNA circulating in the peripheral blood is mostly released by apoptotic cells and necrotic tumor cells but also from extracellular vesicles [7]. cfDNA analysis overcomes the sampling biases inherent to intra-tumor genetic heterogeneity. The modal fragment size for tumor cfDNA and healthy cfDNA is 166 bp, but tumor cfDNA displays an increased proportion of short fragments (100–150 bp) [8]. In cancer patients, only a small portion of cfDNA (usually 0.01–5%) is shed into the blood by tumor cells; this is called ctDNA [9]. Tumor volume of 10 cm^3^ (27 mm in diameter) leads to 0.1% ctDNA in the circulation [10], but cancer type and biological characteristics can also influence the concentration of ctDNA. Therefore, development of ultrasensitive methods to detect 0.01% or less ctDNA in blood plasma is necessary.

Abnormal expansion of clonally derived hematopoietic stem and/or progenitor cells carrying somatic mutations is called clonal hematopoiesis (CH) [11]. CH is associated with an increased risk of hematological malignancies, cardiovascular disease, and greater mortality of non-hematological cancers [12–15]. The most commonly mutated genes in CH are *DNMT3A*, *TET2* and *ASXL1* [16, 17]. In addition, CH is known to lead to false positive results in cfDNA testing, thus complicating the interpretation of liquid biopsy data [18, 19].

Next-generation sequencing (NGS) and digital droplet PCR (ddPCR) are more sensitive mutational analysis techniques. These methods enable detection of cfDNA with somatic mutations and have been used in different types of cancers. NGS-based methods involve targeted [20–22] and untargeted approaches and are well known for their outstanding parallel sequencing ability. Untargeted NGS methods such as whole-genome or whole-exome sequencing have also been used to detect mutants of ctDNA, but at a much higher cost to achieve similar sensitivity. ddPCR can detect known mutants at 0.1% or lower in the blood, and has been used for hotspot mutant detection; it also suitable for the verification of NGS results.

The goals of this study were to evaluate the efficacy and clinical impacts of liquid biopsy on cancer patients and healthy controls using a NGS panel targeting 275 cancer-related genes. We also evaluated CH and germline mutations of patients after analyzing the characteristics of mutants in white blood cells (WBCs) and plasma.

## Methods

### Clinical cohort

We retrospectively reviewed the sequence data from 139 subjects who underwent genetic testing from May 2017 to July 2021. Participants were excluded if they had a blood cancer. Blood samples were collected at 3 months after surgery in early stage patients. Advanced stage patients with were included, regardless of surgery or treatments. We included 92 patients with lung (36), ovarian (27), colorectal (8), breast (5), endometrial (3), gastric (2), renal cell (2), prostate (2), urothelial (1), head and neck (1), hepatocellular (1), neuroendocrine (1), pancreatic (1), cervical (1), or fallopian tube (1) cancer and 47 healthy individuals. This study was approved by the Institutional Review Board of the China Medical University Hospital (CMUH106-REC1–047).

### Sample processing and DNA extraction

Plasma was collected in cell-free DNA collection tubes (Roche, Basel, Switzerland) and separated by centrifugation. Whole blood was centrifuged at 1600×*g* for 20 min at 20 °C. After separating red blood cells and the buffy coat, we centrifuged the plasma a second time at 16,000×*g* for 10 min at 20 °C to remove residual cells. Supernatants were immediately stored at − 80 °C until ready for further processing.

Frozen aliquots of plasma (4–5 mL) were thawed at room temperature, and cfDNA was isolated using a QIAamp Circulating Nucleic Acid Kit (Qiagen, Heidelberg, Germany). Extracted DNA was immediately stored at − 20 °C until further processing. The concentration of purified DNA was measured by fluorometric quantitation using Qubit (Thermo Fisher).

### Next-generation library preparation and sequencing

NGS testing was performed using the QIAseq targeted Human Comprehensive Cancer Panel (Qiagen), which contains 275 genes covering the most commonly occurring mutations in cancer (cat. no. DHS-3501Z). The method has been described in detail in previous studies [23, 24].

### Data analysis

Base calling and quality scoring were performed with an updated implementation of Real-Time Analysis on the NextSeq 500 system. We used bcl2fastq Conversion Software to demultiplex data and convert BCL files to FASTQ files. Sequence reads were processed by read trimming, read aligning, barcode clustering, and gene-specific primer masking. Finally, single nucleotide polymorphisms (SNPs) and small insertion-deletion mutations (INDELs) were called in individual samples using smCounter at the default settings. We used ANNOVAR to annotate variants; in particular, dbSNP and ClinVar, were used to determine whether the variants had been previously identified. Germline mutations with a ≥ 30% allelic fractions (AFs) in both WBC DNA and cfDNA were analyzed.

Several filter procedures were executed after mutation calling. (1) Synonymous variants were filtered out. (2) Variants with low depth (< 500× in cfDNA, 100× in WBC DNA) were filtered out. Variants with < 5 high-quality sequencing reads for cfDNA and 2 high-quality sequencing reads for WBC DNA were removed. (3) An in-house database of 191 cancer patients and 24 healthy individuals was created. Variants were filtered out if present in > 5% of samples in the in-house database and > 1% in dbSNP. The remaining variants were identified as high-confidence somatic mutations.

### Statistical analysis

Nonparametric Mann-Whitney tests were performed to compare ages in different groups. A Kaplan-Meier plot with log-rank test was employed to compare survival among groups. Independent prognostic factors were analyzed by the Cox proportional harzards regression model. Statistical analysis was performed using GraphPad Prism (version 8.0.2; GraphPad Software, San Diego, CA, USA) and SPSS 22.0 (IBM, Armonk. NY, USA). *P* < 0.05 was considered statistically significant.

## Results

### Description of analytical cohort

We obtained 139 peripheral blood samples from 92 patients and 47 healthy individuals. The patient cohort encompassed 15 principal tumor types. The most common tumor type was lung cancer (*n* = 36). Other common types included ovarian cancer (*n* = 27), colorectal cancer (*n* = 8), and breast cancer (*n* = 5). Demographic characteristics of the 139 participants are summarized in Table 1. Detailed information is presented in Additional file 1: Table S1. All plasma samples were sequenced to deep coverage (median, 9804×; range, 1594–43,746×) to ensure high sensitivity for the detection of genomic alterations. The median sequencing depth for WBCs was 944× (range, 105–15,636×).

**Table 1:** General characteristics of participants (*N* = 139)

Variable	Categories	Patient subjects (***N*** = 92) N (%)	Healthy subjects (***N*** = 47) N (%)
Gender	Male	32 (34.78)	30 (63.83)
	Female	60 (65.22)	17 (36.17)
Age	≤45	15 (16.30)	10 (21.28)
	46–60	45 (48.91)	19 (40.43)
	61–75	25 (27.17)	16 (34.04)
	≥76	5 (5.43)	2 (4.26)
	NA	2 (2.17)	NA
Tumor types	Lung	36 (39.13)	NA
	Ovarian	27 (29.35)	NA
	Colorectal	8 (8.70)	NA
	Breast	5 (5.43)	NA
	Endometrial	3 (3.26)	NA
	Gastric	2 (2.17)	NA
	Prostate	2 (2.17)	NA
	Renal cell	2 (2.17)	NA
	Head and neck	1 (1.09)	NA
	Liver	1 (1.09)	NA
	Urothelial	1 (1.09)	NA
	Other	4 (4.35)	NA

### Some cfDNA mutations originate from CH variants in WBCs

**Fig. 1:**
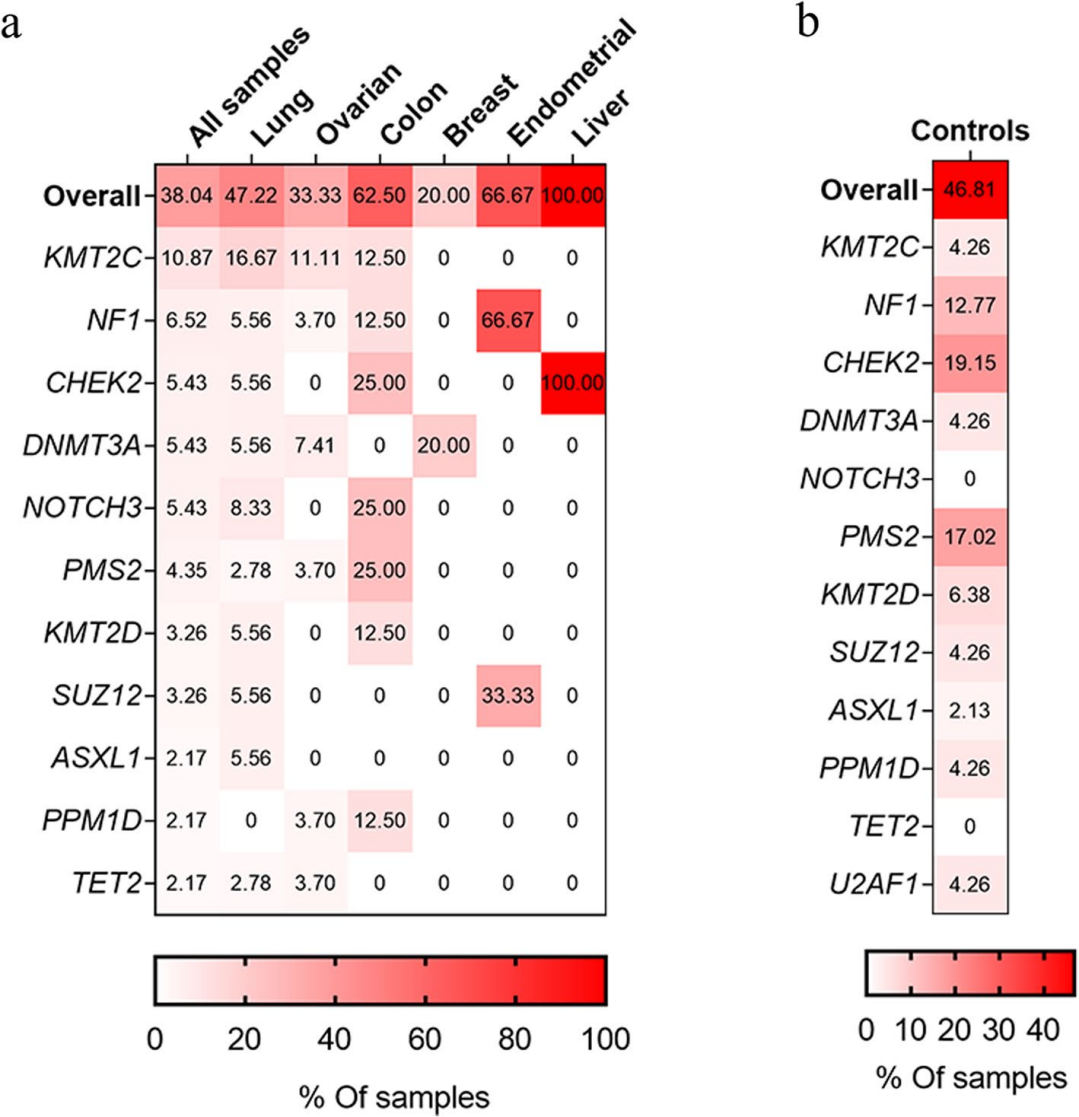
Identifying CH variants in plasma cfDNA via matched WBC sequencing. **a** Percentage of plasma samples with identified CH variants in different cancer types. The first row indicates the overall percentage of samples with CH variants in different cancer types. The remaining rows indicate the percentage of samples with CH variants in recurrent and canonical genes. **b** Percentage of plasma samples with identified CH variants in controls

**Fig. 2:**
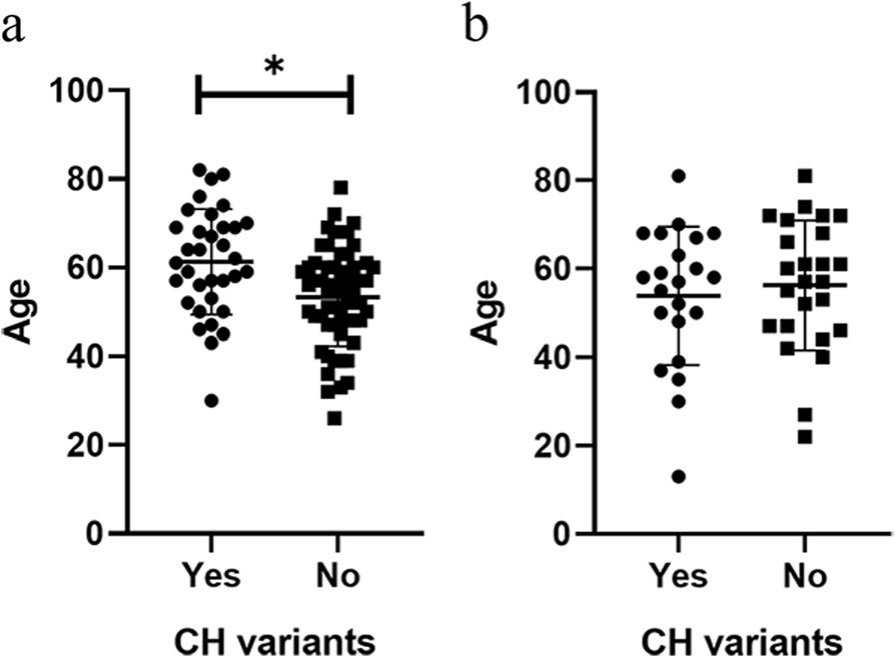
Age of **a** patients and **b** healthy controls with and without CH variants. Statistical analysis was performed using the Mann-Whitney test

Ultradeep sequencing was performed for WBCs of the 92 cancer patients to characterize the sources of the cfDNA mutations detected in plasma. A total of 138 mutations detected from 35 samples of plasma were also detected in WBCs, suggesting a hematopoietic origin (Additional file 2: Table S2). *KMT2C* (10.87%, 10/92), *NF1* (6.52%, 6/92), *CHEK2*, *DNMT3A*, *NOTCH3* (5.43%, 5/92), *PMS2* (4.35%, 4/92), *KMT2D* (3.26%, 3/92) and *SUZ12* (3.26%, 3/92) were the most recurrent. For *ASXL1*, *BCR*, *CUX1*, *FANCD2*, *GATA2*, *MYCL*, *PPM1D*, *SOX9*, *TERT*, *TET2*, and *TSC2*, a mutation of each gene was found in two patients (2.17%, 2/92) (Fig. 1a). Among the 15 canonical genes associated with CH, our cancer patients had mutations in *CHEK2*, *DNMT3A*, *ASXL1*, *PPM1D*, and *TET2* only (Fig. 1a). Furthermore, cancer patients with CH variants were significantly older than those without CH variants in cfDNA (61 vs. 53 years, *p* = 0.0024) (Fig. 2a). We also examined the association between the CH variants and stage of cancer patients. The results showed that the CH variants are not associated with cancer’s stage (*p* = 0.3058) (Additional file 3: Table S3).

In healthy individuals, 66 mutations detected from 22 plasma samples were also detected in WBCs, suggesting their hematopoietic origin (Additional file 4: Table S4). Mutations in *CHEK2* (19.15%, 9/47), *PMS2* (17.02%, 8/47), *NF1* (12.77%, 6/47), *KMT2D* (6.38%, 3/47), *BCR*, *DNMT3A*, *FANCD2*, *KMT2C*, *PPM1D*, *RAD50*, *SUZ12*, and *U2AF1* (4.26%, 2/47) were the most recurrent (Fig. 1b). The remaining mutations of CH-related genes were identified in one sample. Mutations of five (*CHEK2*, *DNMT3A*, *PPM1D*, *U2AF1*, and *ASXL1*) of 15 canonical CH genes were found in the healthy subjects (Fig. 1b). No statistical differences were observed in the age of the healthy subjects in the cohort with at least one CH-related mutation and in that without a CH-related mutation (54 vs. 56 years, *p* = 0.5933) (Fig. 2b).

### Mutation landscape of pan-cancer ctDNA

Twenty-one cancer patients (22.83%, 21/92) had a somatic mutation(s) classified as pathogenic (P)/likely pathogenic (LP) in the ClinVar database (Additional file 5: Table S5). The most frequently mutated gene was *TP53* (9/92, 9.78%), followed by *KMT2D*, *NF1*, *PIK3CA*, and *SOX2*, which were each found in three separate cases (3/92, 3.26%) and *CTNNB1*, *FGFR2*, *MSH6*, and *PTEN,* which were each found in two separate cases (2/92, 2.17%). *APC*, *BRAF*, *BRCA2*, *EGFR*, *ERBB2*, *IDH1*, *KRAS*, and *NTRK1* were each found in one case (1/92, 1.09%).

**Fig. 3:**
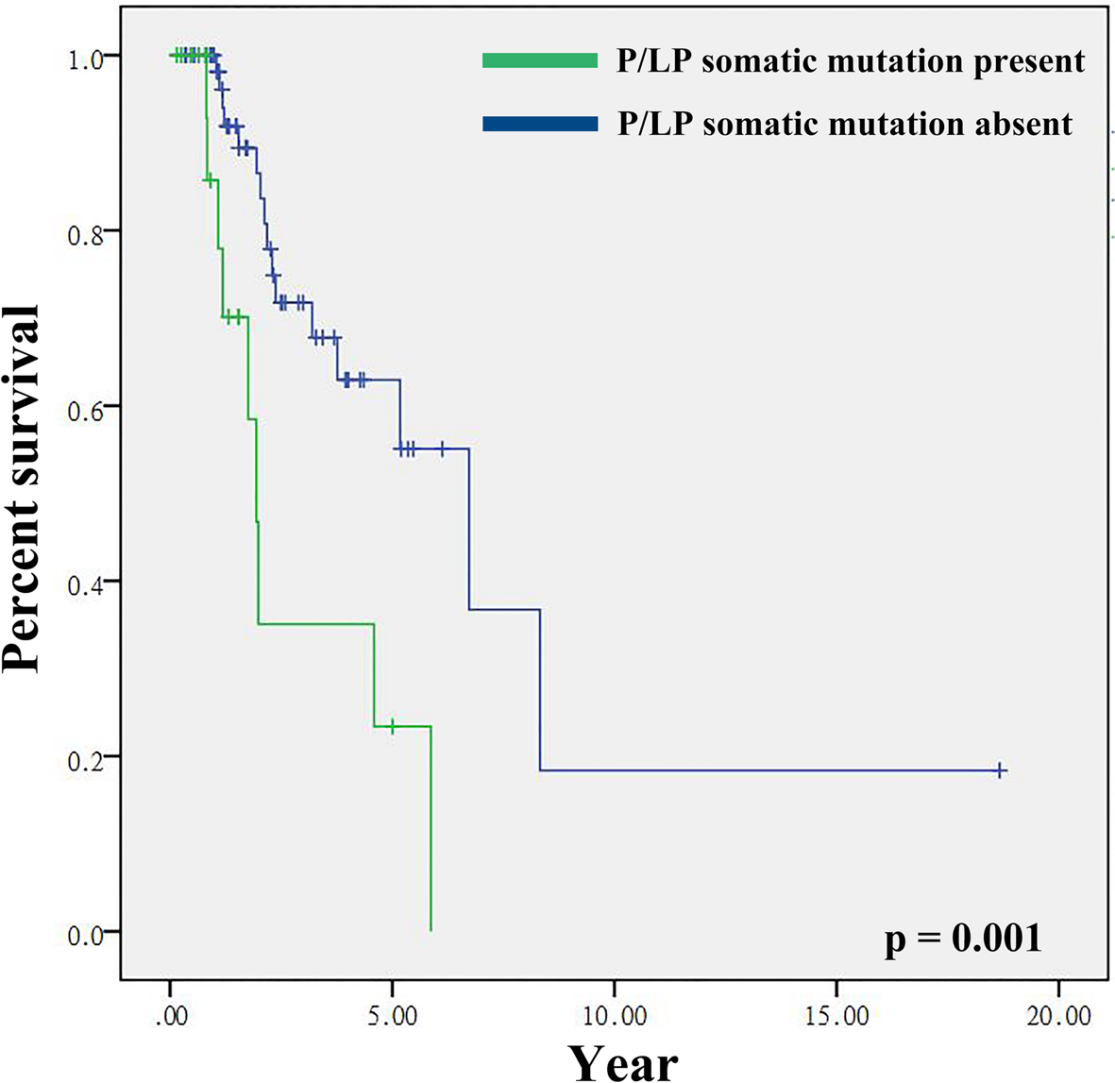
Kaplan-Meier curve in patients with and without mutations in P/LP somatic cancer-related genes

We also compared the overall survival (OS) of cancer patients with versus without a somatic P/LP variant in ctDNA. OS was better in those without P/LP cancer-related gene mutations, as compared to those with mutations (7.42 vs. 2.87 years, respectively); this association was statistically significant (*p* = 0.001; Fig. 3). Multivariate analysis that incorporated independent prognostic factors of CH-related mutation, germline mutation, and tumor stage revealed that the presence of P/LP somatic mutations was significantly correlated with OS (*p* = 0.022) (Table 2).

**Table 2:** Multivariate analysis (Cox regression) of independent prognostic factors in patients with cancer

Variables		Harzard Ratio	95% CI	***p***-value
CH-related mutations	–	1	0.512–2.778	0.684
	+	1.192		
Somatic P/LP mutations	–	1	1.166–7.011	0.022
	+	2.859		
Germline P/LP mutations	–	1	0.054–2.437	0.297
	+	0.364		
Tumor stage	I and II	1	0.509–14.731	2.737
	III and IV	2.737		

One healthy individual (2.13%, 1 of 47) had a somatic mutation of the *MYC* gene classified as P/LP in the ClinVar database (Additional file 6: Table S6). The clinical impact of this variant will require close observation and follow-up.

### Frequency of germline P/LP mutations detected in cfDNA

Seven cancer patients (7.61%, 7/92) had an evaluable candidate germline variant(s) with a variant allele frequency (VAF) between 30 and 60%, irrespective of pathogenicity on ctDNA analysis. The germline variants identified were *MSH2* p.R711X, *BRCA1* p.T1691K, *MUTYH* p.R95W, *RAD50* p.L719fs, *BRCA2* p.T587fs, *BRIP1* p.W448X, and *MPL* c.981-1G > C (Additional file 7: Table S7). Of 7 patients with a germline mutation, two (28.57%) had a family history with cancer.

One healthy individual (2.13%, 1/47) had a candidate germline variant identified as *NOTCH3* p.R544C (Additional file 8: Table S8). This variant was present at a VAF of 47.41% (247/521) in cfDNA and 49.05% (258/526) in matched buffy coat.

### Case presentation

**Fig. 4:**
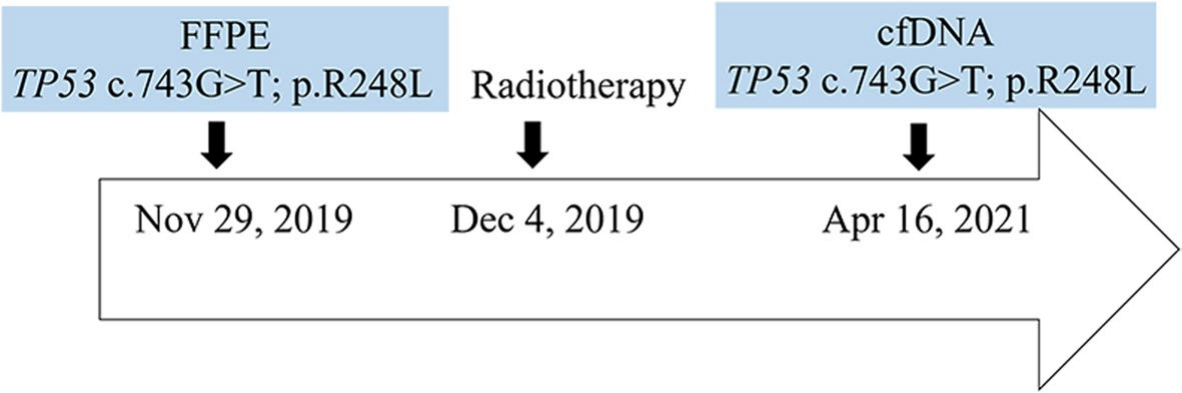
Timeline of events from surgery and cfDNA sequencing of the patient

We only have nine cases involving both FFPE and liquid biopsy samples (Additional file 9: Table S9). For example, we compared the concordance between FFPE and ctDNA genomic profiling of one lung cancer patient. *TP53* p.R248L P mutation was found in two different types samples. This patient receive radiotherapy during this period (Fig. 4). The result indicated that *TP53* mutation may induce resistance to certain cancer therapy.

## Discussion

Herein, we report a study of non-invasive ctDNA detection for Taiwanese cancer patients and healthy individuals. We analyzed the detected variants and further characterized them as CH (Additional file 10: Fig. S1), somatic, or germline variants (Additional file 11: Fig. S2). Overall, 22.83% of cancer patients harbored P/LP somatic mutations. As expected, a lower frequency (2.13%) in healthy individuals was observed. The majority of cancer patients (58%) had ≥1 ctDNA alteration(s) [25]. In the present study, somatic mutations were only evaluated in the ClinVar database as P/LP; variants of undetermined significance, synonymous, or further analyzed by prediction tools were excluded. As a result, the detection rate of somatic alterations in our study was lower than that of other published studies. One of the 47 healthy individuals carried at least one P/LP somatic mutation in our study, in contrast with another study [19]. ctDNA analysis of this person using NGS or ddPCR is recommended to detect the variant change, and more strict clinical study may be needed if the plasma concentration of the variant is elevated.

We also identified seven P/LP germline variants in seven cancer-related genes (*BRCA1*, *BRCA2*, *BRIP1*, *MPL*, *MSH2*, *MUTYH*, and *RAD50*) in 7.61% (7/92) of cancer patients. These germline mutations were detected in three ovarian, two lung, one cervical, and one endometrial cancer patient; most of the mutations produced stop codons, frameshifts, or aberrant splicing resulting in loss of the protein. Thus these mutations are likely to influence greatly or inhibit protein function. Many studies have explored the association between germline variants and somatic aberrations [26, 27], and carriers of germline variants in our study are already known as high penetrance mutants for cancer development, e.g., P/LP germline mutations in 12 genes (*BARD1*, *BRCA1*, *BRCA2*, *BRIP1*, *PALB2*, *RAD51C*, *RAD51D*, *MSH2*, *MLH1*, *PMS2*, *MSH6*, and *EPCAM*) are known or suspected to increase the risk of ovarian cancer [28]. Among these ovarian cancer susceptibility genes, we identified P/LP germline variants in *BRCA1* and *MSH2* in our ovarian cancer cohort. *MUTYH* germline mutations are best known for their role in colorectal cancer. Win et al. reported that biallelic germline *MUTYH* mutations confer a 14% risk of ovarian cancer by age 70 [29]. In the current study, we identified a *MUTYH* germline mutation in one ovarian cancer patient. A previous study in 36,813 Chinese lung cancer patients, focusing on eight key lung cancer driver genes (*EGFR*, *ALK*, *MET*, *KRAS*, *ERBB2*, *ROS1*, *RET*, and *BRAF*), revealed a prevalence of 0.03% for P/LP germline mutations [30]. However, we did not find germline mutations in these genes. In our lung cancer patient cohort, *BRIP1* (p.W448X) and *MPL* (c.981-1G > C) germline mutations were detected. Germline mutations in *BRIP1* and *MPL* were associated with increased ovarian cancer risks and hereditary thrombocytosis, respectively [31, 32]. Liu et al. observed *BRIP1* LP germline mutations (p.M1V and p.T977fs) in lung cancer [33]. However, the spectrum of mutation (p.W448X) is different to that reported by Liu et al. *RAD50* germline mutation (p.L719fs), identified by Fan et al. in breast cancer patients, is consistent with our analysis of cervical cancer patient [34]. Germline mutations in *BRCA* have been associated with cases of endometrial cancer, mainly in *BRCA1* [35]. In the present study, we identified a *BRCA2* germline mutation, p.T587fs, in patient with endometrial cancer. From these results, we recommend familial cancer consultations for the family members of these patients.

We identified one LP germline mutation, p.R544C, in *NOTCH3* in healthy individuals. Germline mutation has not been previously described in the *NOTCH3* gene. The clinical significance of this variant warrants further study, and we recommend that this individual be closely monitored to allow for early detection of cancer if necessary.

We found that 38.04% of patients carried CH mutations, which differs slightly from other studies; we suggest that the rate is dependent on the materials and methods used. Highly sensitive cfDNA approaches have identified CH mutations in 89.5% of patients with cancer and 83% of controls without cancer [17]. Chan et al. detected CH-related mutations in 29% (11/38) of colorectal cancer patients [36]. A recent study conducted by Zhang et al. found that 14.0% (1861/13,333) of cancer patients harbored CH variants in plasma samples [37]. A different NGS panel and sequencing paired plasma-WBCs could lead to differing prevalence of CH detection in cfDNA. Liu et al. showed the ineffectiveness of distinguishing CH mutations of low VAF (≦0.1%) from tumor-derived mutations using conventional NGS of blood cell DNA [38]. We set our minimum VAF requirements to > 1%; thus, some CH mutations may have been missed, which may result in a slightly lower occurrence rate in our data.

Age-associated mutations including cytosine deamination, DNA double-strand breaks, polymerase error, and structure rearrangements of chromosomes are common. Adult humans have hematopoietic stem cells (HSCs) about 50,000 to 200,000, and harbor up to 1.4 million protein coding mutations in HSC pool by age 70, and these mutations may cause clonal expansions [39]. This reason can be used to explain our results that older patients have more frequent CH-related mutations.

CH can lead to blood cancers, therefore CH mutations detected in myelodysplastic syndrome and acute myeloid leukemia is important [40]. In patients with solid tumors, matched cfDNA-WBC sequencing can be used to distinguish CH somatic mutations from those in the solid tumor cells. When CH mutations are actionable alterations, it may lead to erroneous treatment recommendations. Early-stage cancers [41], minimal residual disease [42], and intra- and intertumoral heterogeneity [43] may have a low VAF, similar to CH, and these results may lead to false negatives in the clinical setting. To address this, we sequenced the buffy coat of blood, and were able to differentiate CH from the above-mentioned conditions. In patients with cancer, CH is a common occurrence, and associated with aging, smoking, and radiation therapy [12]. CH has been linked to decreased overall survival, including greater risk of cardiovascular mortality [13]. Whether CH can be applied as the prognosis biomarker for solid tumor need further study.

Liquid biopsy has many clinical impacts. Recent studies have shown that detected positive cases have poorer survival than detected negative cases including therapeutic response and prognosis [44–48]. This is consistent with our findings. Our results showed that the presence of P/LP variants in cancer-related genes predicted shorter OS in patients (2.87 vs. 7.42 years, *p* = 0.001). Multivariate analysis adjusted for CH-related mutation, germline mutation, and tumor stage also indicated that somatic mutations correlate significantly with OS (*p* = 0.022). We also examined the effect of P/LP somatic mutation in lung (36 cases) and ovarian (27 cases) cancer patients separately. But, there was no statistically significant difference between the two groups with respect to P/LP somatic mutation in two different cancer types, which may be due to small number of these cancers, and different treatment history. The appearance of P/LP in the results of liquid biopsy has strong correlation with patients prognosis is confirmed by many studies that including many types of cancers. Our study showed P/LP influencing the survival of unselected cancer types.

## Conclusions

In summary, the present study identified the mutational spectra of pan-cancer in a Taiwanese population. ctDNA analysis has important clinical impacts. In addition, matched cfDNA-WBC sequencing is important for accurate variant interpretation.

## Supplementary Information

**Additional file 1: Table S1**. Clinical and pathological characteristics of the study cohort of cancer patients.

**Additional file 2: Table S2**. cfDNA CH-related variants list in cancer patients.

**Additional file 3: Table S3**. Correlation between cancer stage and CH-related variants.

**Additional file 4: Table S4**. cfDNA CH-related variants list in healthy individuals.

**Additional file 5: Table S5**. cfDNA P/LP somatic mutations list in cancer patients.

**Additional file 6: Table S6**. cfDNA P/LP somatic mutations list in healthy individuals.

**Additional file 7: Table S7**. cfDNA P/LP germline mutations list in cancer patients.

**Additional file 8: Table S8**. cfDNA P/LP germline mutations list in healthy individuals.

**Additional file 9: Table S9**. Characteristics of next-generation sequencing outcomes of FFPE and cfDNA in different time.

**Additional file 10: Figure S1**. Oncoprint showing the distribution of CH genes in cancer patients.

**Additional file 11: Figure S2**. Oncoprint showing the distribution of genomic alterations in both somatic and germline genomes in cancer patients.

## Data Availability

The datasets generated and analyzed during the current study are not publicly available since proper ethical permission for open data access has not been obtained, but are available from the corresponding author on reasonable request.

## Abbreviations

CH: Clonal hematopoiesis
P: Pathogenic
LP: Likely pathogenic
OS: Overall survival
CTCs: Circulating tumor cells
ctDNA: Circulating tumor-derived DNA
cfDNA: Cell-free DNA
NGS: Next-generation sequencing
ddPCR: Digital droplet PCR
WBCs: White blood cells
SNPs: Single nucleotide polymorphisms
INDELs: Insertion-deletion mutations
AFs: Allelic fractions
VAF: Variant allele frequency
HSCs: Hematopoietic stem cells

## References

[1] Heidrich I, Ackar L, Mossahebi Mohammadi P, Pantel K (2021). Liquid biopsies: potential and challenges. Int J Cancer.

[2] Cheng FT, Lapke N, Wu CC, Lu YJ, Chen SJ, Yu PN, Liu YT, Tan KT (2019). Liquid biopsy detects relapse five months earlier than regular clinical follow-up and guides targeted treatment in breast cancer. Case Rep Oncol Med.

[3] Kalluri R, LeBleu VS. The biology, function, and biomedical applications of exosomes. Science. 2020;367(6478):eaau6977. 10.1126/science.aau6977 PMC7717626 32029601

[4] Anfossi S, Fu X, Nagvekar R, Calin GA (2018). MicroRNAs, regulatory messengers inside and outside cancer cells. Adv Exp Med Biol.

[5] Best MG, Wesseling P, Wurdinger T (2018). Tumor-educated platelets as a noninvasive biomarker source for cancer detection and progression monitoring. Cancer Res.

[6] Mandel P (1948). Les acides nucleiques du plasma sanguin chez 1 homme. C R Seances Soc Biol Fil.

[7] Thierry AR, El Messaoudi S, Gahan PB, Anker P, Stroun M (2016). Origins, structures, and functions of circulating DNA in oncology. Cancer Metastasis Rev.

[8] Guo J, Ma K, Bao H, Ma X, Xu Y, Wu X, Shao YW, Jiang M, Huang J (2020). Quantitative characterization of tumor cell-free DNA shortening. BMC Genomics.

[9] Pantel K, Alix-Panabieres C (2019). Liquid biopsy and minimal residual disease - latest advances and implications for cure. Nat Rev Clin Oncol.

[10] Abbosh C, Birkbak NJ, Wilson GA, Jamal-Hanjani M, Constantin T, Salari R, Le Quesne J, Moore DA, Veeriah S, Rosenthal R (2017). Phylogenetic ctDNA analysis depicts early-stage lung cancer evolution. Nature.

[11] Park SJ, Bejar R (2020). Clonal hematopoiesis in cancer. Exp Hematol.

[12] Coombs CC, Zehir A, Devlin SM, Kishtagari A, Syed A, Jonsson P, Hyman DM, Solit DB, Robson ME, Baselga J (2017). Therapy-related clonal hematopoiesis in patients with non-hematologic cancers is common and associated with adverse clinical outcomes. Cell Stem Cell.

[13] Jaiswal S, Natarajan P, Silver AJ, Gibson CJ, Bick AG, Shvartz E, McConkey M, Gupta N, Gabriel S, Ardissino D (2017). Clonal hematopoiesis and risk of atherosclerotic cardiovascular disease. N Engl J Med.

[14] Jaiswal S, Fontanillas P, Flannick J, Manning A, Grauman PV, Mar BG, Lindsley RC, Mermel CH, Burtt N, Chavez A (2014). Age-related clonal hematopoiesis associated with adverse outcomes. N Engl J Med.

[15] Genovese G, Kahler AK, Handsaker RE, Lindberg J, Rose SA, Bakhoum SF, Chambert K, Mick E, Neale BM, Fromer M (2014). Clonal hematopoiesis and blood-cancer risk inferred from blood DNA sequence. N Engl J Med.

[16] Ptashkin RN, Mandelker DL, Coombs CC, Bolton K, Yelskaya Z, Hyman DM, Solit DB, Baselga J, Arcila ME, Ladanyi M (2018). Prevalence of clonal hematopoiesis mutations in tumor-only clinical genomic profiling of solid tumors. JAMA Oncol.

[17] Razavi P, Li BT, Brown DN, Jung B, Hubbell E, Shen R, Abida W, Juluru K, De Bruijn I, Hou C (2019). High-intensity sequencing reveals the sources of plasma circulating cell-free DNA variants. Nat Med.

[18] Spoor J, Eyck BM, Atmodimedjo PN, Jansen M, Helmijr JCA, Martens JWM, van der Wilk BJ, van Lanschot JJB, Dinjens WNM (2021). Liquid biopsy in esophageal cancer: a case report of false-positive circulating tumor DNA detection due to clonal hematopoiesis. Ann Transl Med.

[19] Hu Y, Ulrich BC, Supplee J, Kuang Y, Lizotte PH, Feeney NB, Guibert NM, Awad MM, Wong KK, Janne PA (2018). False-positive plasma genotyping due to clonal hematopoiesis. Clin Cancer Res.

[20] Forshew T, Murtaza M, Parkinson C, Gale D, Tsui DW, Kaper F, Dawson SJ, Piskorz AM, Jimenez-Linan M, Bentley D (2012). Noninvasive identification and monitoring of cancer mutations by targeted deep sequencing of plasma DNA. Sci Transl Med.

[21] Kinde I, Wu J, Papadopoulos N, Kinzler KW, Vogelstein B (2011). Detection and quantification of rare mutations with massively parallel sequencing. Proc Natl Acad Sci U S A.

[22] Newman AM, Bratman SV, To J, Wynne JF, Eclov NC, Modlin LA, Liu CL, Neal JW, Wakelee HA, Merritt RE (2014). An ultrasensitive method for quantitating circulating tumor DNA with broad patient coverage. Nat Med.

[23] Chang YS, Fang HY, Hung YC, Ke TW, Chang CM, Liu TY, Chen YC, Chao DS, Huang HY, Chang JG (2018). Correlation of genomic alterations between tumor tissue and circulating tumor DNA by next-generation sequencing. J Cancer Res Clin Oncol.

[24] Chang YS, Tu SJ, Chen YC, Liu TY, Lee YT, Yen JC, Fang HY, Chang JG (2021). Mutation profile of non-small cell lung cancer revealed by next generation sequencing. Respir Res.

[25] Schwaederle M, Husain H, Fanta PT, Piccioni DE, Kesari S, Schwab RB, Patel SP, Harismendy O, Ikeda M, Parker BA (2016). Use of liquid biopsies in clinical oncology: pilot experience in 168 patients. Clin Cancer Res.

[26] Ramroop JR, Gerber MM, Toland AE (2019). Germline variants impact somatic events during tumorigenesis. Trends Genet.

[27] Chatrath A, Ratan A, Dutta A (2021). Germline variants that affect tumor progression. Trends Genet.

[28] Somasegar S, Weiss AS, Norquist BM, Khasnavis N, Radke M, Manhardt E, Pennil C, Pennington KP, Eckert MA, Chryplewicz A (2021). Germline mutations in black patients with ovarian, fallopian tube and primary peritoneal carcinomas. Gynecol Oncol.

[29] Win AK, Reece JC, Dowty JG, Buchanan DD, Clendenning M, Rosty C, Southey MC, Young JP, Cleary SP, Kim H (2016). Risk of extracolonic cancers for people with biallelic and monoallelic mutations in MUTYH. Int J Cancer.

[30] Yang J, Li H, Li B, Li W, Guo Q, Hu L, Song Z, Zhou B (2021). Profiling oncogenic Germline mutations in unselected Chinese lung Cancer patients. Front Oncol.

[31] Ramus SJ, Song H, Dicks E, Tyrer JP, Rosenthal AN, Intermaggio MP, et al. Germline mutations in the BRIP1, BARD1, PALB2, and NBN genes in women with ovarian cancer. J Natl Cancer Inst. 2015;107(11):djv214. 10.1093/jnci/djv214 PMC4643629 26315354

[32] Ding J, Komatsu H, Wakita A, Kato-Uranishi M, Ito M, Satoh A, Tsuboi K, Nitta M, Miyazaki H, Iida S (2004). Familial essential thrombocythemia associated with a dominant-positive activating mutation of the c-MPL gene, which encodes for the receptor for thrombopoietin. Blood.

[33] Liu M, Liu X, Suo P, Gong Y, Qu B, Peng X, Xiao W, Li Y, Chen Y, Zeng Z (2020). The contribution of hereditary cancer-related germline mutations to lung cancer susceptibility. Transl Lung Cancer Res.

[34] Fan C, Zhang J, Ouyang T, Li J, Wang T, Fan Z, Fan T, Lin B, Xie Y (2018). RAD50 germline mutations are associated with poor survival in BRCA1/2-negative breast cancer patients. Int J Cancer.

[35] Matanes E, Volodarsky-Perel A, Eisenberg N, Rottenstreich M, Yasmeen A, Mitric C, Lau S, Salvador S, Gotlieb WH, Kogan L (2021). Endometrial cancer in Germline BRCA mutation carriers: a systematic review and meta-analysis. J Minim Invasive Gynecol.

[36] Chan HT, Nagayama S, Chin YM, Otaki M, Hayashi R, Kiyotani K, Fukunaga Y, Ueno M, Nakamura Y, Low SK (2020). Clinical significance of clonal hematopoiesis in the interpretation of blood liquid biopsy. Mol Oncol.

[37] Zhang Y, Yao Y, Xu Y, Li L, Gong Y, Zhang K, Zhang M, Guan Y, Chang L, Xia X (2021). Pan-cancer circulating tumor DNA detection in over 10,000 Chinese patients. Nat Commun.

[38] Liu J, Chen X, Wang J, Zhou S, Wang CL, Ye MZ, Wang XY, Song Y, Wang YQ, Zhang LT (2019). Biological background of the genomic variations of cf-DNA in healthy individuals. Ann Oncol.

[39] Jaiswal S, Ebert BL. Clonal hematopoiesis in human aging and disease. Science. 2019;366(6465):eaan4673. 10.1126/science.aan4673 PMC8050831 31672865

[40] Pasca S, Gondek LP (2021). Clonal hematopoiesis and bone marrow failure syndromes. Best Pract Res Clin Haematol.

[41] Fiala C, Diamandis EP (2018). Utility of circulating tumor DNA in cancer diagnostics with emphasis on early detection. BMC Med.

[42] Rolfo C, Cardona AF, Cristofanilli M, Paz-Ares L, Diaz Mochon JJ, Duran I, Raez LE, Russo A, Lorente JA, Malapelle U (2020). Challenges and opportunities of cfDNA analysis implementation in clinical practice: perspective of the International Society of Liquid Biopsy (ISLB). Crit Rev Oncol Hematol.

[43] Nelson AC, Boone J, Cartwright D, Thyagarajan B, Kincaid R, Lambert AP, Karnuth K, Henzler C, Yohe S (2018). Optimal detection of clinically relevant mutations in colorectal carcinoma: sample pooling overcomes intra-tumoral heterogeneity. Mod Pathol.

[44] Oliveira KCS, Ramos IB, Silva JMC, Barra WF, Riggins GJ, Palande V, Pinho CT, Frenkel-Morgenstern M, Santos SEB, Assumpcao PP (2020). Current perspectives on circulating tumor DNA, precision medicine, and personalized clinical management of cancer. Mol Cancer Res.

[45] Mack PC, Banks KC, Espenschied CR, Burich RA, Zill OA, Lee CE, Riess JW, Mortimer SA, Talasaz A, Lanman RB (2020). Spectrum of driver mutations and clinical impact of circulating tumor DNA analysis in non-small cell lung cancer: analysis of over 8000 cases. Cancer.

[46] Cullinane C, Fleming C, O'Leary DP, Hassan F, Kelly L, O'Sullivan MJ, Corrigan MA, Redmond HP (2020). Association of circulating tumor DNA with disease-free survival in breast cancer: a systematic review and meta-analysis. JAMA Netw Open.

[47] Thusgaard CF, Korsholm M, Koldby KM, Kruse TA, Thomassen M, Jochumsen KM (2021). Epithelial ovarian cancer and the use of circulating tumor DNA: a systematic review. Gynecol Oncol.

[48] Jones RP, Pugh SA, Graham J, Primrose JN, Barriuso J (2021). Circulating tumour DNA as a biomarker in resectable and irresectable stage IV colorectal cancer; a systematic review and meta-analysis. Eur J Cancer.

