# Supplementary material for: Clinical application of liquid biopsy in cancer patients
Source: BMC Cancer. 2022 Apr 15;22:413. doi: 10.1186/s12885-022-09525-0 (PMC9011972; doi:10.1186/s12885-022-09525-0)
Supplement: Supplementary file 1 — Additional file 1: Table S1. Clinical and pathological characteristics of the study cohort of cancer patients. [file 12885_2022_9525_MOESM1_ESM.docx]

| Sample_ID | Cancer types | Chrom | start_pos | end_pos | ref | alt | Variants | Depth_and_Ratio in plasma | Depth_and_Ratio in WBCs | Allele_Freq | clinvar | avsnp | Type | HGVS |
| --- | --- | --- | --- | --- | --- | --- | --- | --- | --- | --- | --- | --- | --- | --- |
| F711130217 | Ovarian | chr7 | 6043666 | 6043666 | C | T | chr7:g.6043666C>T (NC_000007.13) | 12/554 (2.17%) | 7/106 (6.60%) | 0.0001 |  | rs772216832 | nonsynonymous SNV | PMS2:NM_000535:c.187G>A:NP_000526:p.V63M\|PMS2:NM_001322006:c.187G>A:NP_001308935:p.V63M\|PMS2:NM_001322014:c.187G>A:NP_001308943:p.V63M |
| F712070229 | Lung | chr12 | 49426592 | 49426592 | A | G | chr12:g.49426592A>G (NC_000012.11) | 14/963 (1.45%) | 32/1876 (1.71%) |  |  |  | nonsynonymous SNV | KMT2D:NM_003482:c.11896T>C:NP_003473:p.F3966L |
| F712070229 | Lung | chr12 | 49445563 | 49445563 | T | G | chr12:g.49445563T>G (NC_000012.11) | 38/702 (5.41%) | 30/801 (3.75%) |  |  |  | nonsynonymous SNV | KMT2D:NM_003482:c.1903A>C:NP_003473:p.M635L |
| F712070229 | Lung | chr12 | 49446094 | 49446094 | T | G | chr12:g.49446094T>G (NC_000012.11) | 19/1307 (1.45%) | 47/2103 (2.23%) |  |  |  | nonsynonymous SNV | KMT2D:NM_003482:c.1372A>C:NP_003473:p.T458P |
| F712070229 | Lung | chr16 | 2138243 | 2138243 | C | T | chr16:g.2138243C>T (NC_000016.9) | 25/915 (2.73%) | 20/1152 (1.74%) | 0.0001 | Uncertain_significance | rs761618860 | nonsynonymous SNV | TSC2:NM_000548:c.5176C>T:NP_000539:p.H1726Y\|TSC2:NM_001077183:c.4975C>T:NP_001070651:p.H1659Y\|TSC2:NM_001114382:c.5107C>T:NP_001107854:p.H1703Y\|TSC2:NM_001318827:c.4867C>T:NP_001305756:p.H1623Y\|TSC2:NM_001318829:c.4831C>T:NP_001305758:p.H1611Y\|TSC2:NM_001318831:c.4444C>T:NP_001305760:p.H1482Y\|TSC2:NM_001318832:c.5008C>T:NP_001305761:p.H1670Y |
| F712070229 | Lung | chr16 | 2138244 | 2138244 | A | T | chr16:g.2138244A>T (NC_000016.9) | 20/850 (2.35%) | 11/1033 (1.06%) |  |  |  | nonsynonymous SNV | TSC2:NM_000548:c.5177A>T:NP_000539:p.H1726L\|TSC2:NM_001077183:c.4976A>T:NP_001070651:p.H1659L\|TSC2:NM_001114382:c.5108A>T:NP_001107854:p.H1703L\|TSC2:NM_001318827:c.4868A>T:NP_001305756:p.H1623L\|TSC2:NM_001318829:c.4832A>T:NP_001305758:p.H1611L\|TSC2:NM_001318831:c.4445A>T:NP_001305760:p.H1482L\|TSC2:NM_001318832:c.5009A>T:NP_001305761:p.H1670L |
| F712070229 | Lung | chr16 | 2138247 | 2138247 | A | G | chr16:g.2138247A>G (NC_000016.9) | 28/842 (3.33%) | 19/1076 (1.77%) |  |  |  | nonsynonymous SNV | TSC2:NM_000548:c.5180A>G:NP_000539:p.H1727R\|TSC2:NM_001077183:c.4979A>G:NP_001070651:p.H1660R\|TSC2:NM_001114382:c.5111A>G:NP_001107854:p.H1704R\|TSC2:NM_001318827:c.4871A>G:NP_001305756:p.H1624R\|TSC2:NM_001318829:c.4835A>G:NP_001305758:p.H1612R\|TSC2:NM_001318831:c.4448A>G:NP_001305760:p.H1483R\|TSC2:NM_001318832:c.5012A>G:NP_001305761:p.H1671R |
| F712070229 | Lung | chr17 | 29528142 | 29528142 | T | C | chr17:g.29528142T>C (NC_000017.10) | 10/944 (1.06%) | 75/4126 (1.82%) |  |  |  | nonsynonymous SNV | NF1:NM_000267:c.1150T>C:NP_000258:p.F384L\|NF1:NM_001042492:c.1150T>C:NP_001035957:p.F384L\|NF1:NM_001128147:c.1150T>C:NP_001121619:p.F384L |
| F712070229 | Lung | chr17 | 29541476 | 29541476 | C | T | chr17:g.29541476C>T (NC_000017.10) | 34/875 (3.89%) | 133/5057 (2.63%) |  |  |  | nonsynonymous SNV | NF1:NM_000267:c.1400C>T:NP_000258:p.T467I\|NF1:NM_001042492:c.1400C>T:NP_001035957:p.T467I\|NF1:NM_001128147:c.1400C>T:NP_001121619:p.T467I |
| F712070229 | Lung | chr17 | 29554589 | 29554589 | C | T | chr17:g.29554589C>T (NC_000017.10) | 81/561 (14.44%) | 405/3053 (13.27%) |  | Uncertain_significance | | nonsynonymous SNV | NF1:NM_000267:c.2374C>T:NP_000258:p.L792F\|NF1:NM_001042492:c.2374C>T:NP_001035957:p.L792F |
| F712070229 | Lung | chr17 | 30267331 | 30267331 | C | T | chr17:g.30267331C>T (NC_000017.10) | 15/559 (2.68%) | 73/3385 (2.16%) |  |  | rs2627175 | stopgain | SUZ12:NM_001321207:c.301C>T:NP_001308136:p.R101X\|SUZ12:NM_015355:c.301C>T:NP_056170:p.R101X |
| F712070229 | Lung | chr17 | 30303572 | 30303572 | C | T | chr17:g.30303572C>T (NC_000017.10) | 14/790 (1.77%) | 51/4852 (1.05%) |  |  | rs372162318 | stopgain | SUZ12:NM_001321207:c.787C>T:NP_001308136:p.R263X\|SUZ12:NM_015355:c.856C>T:NP_056170:p.R286X |
| F712070229 | Lung | chr17 | 70119704 | 70119704 | A | C | chr17:g.70119704A>C (NC_000017.10) | 13/670 (1.94%) | 16/704 (2.27%) |  |  |  | nonsynonymous SNV | SOX9:NM_000346:c.706A>C:NP_000337:p.T236P |
| F712070229 | Lung | chr17 | 70120038 | 70120038 | A | C | chr17:g.70120038A>C (NC_000017.10) | 21/789 (2.66%) | 31/1659 (1.87%) |  |  |  | nonsynonymous SNV | SOX9:NM_000346:c.1040A>C:NP_000337:p.Q347P |
| F712070229 | Lung | chr17 | 70120041 | 70120041 | A | C | chr17:g.70120041A>C (NC_000017.10) | 18/726 (2.48%) | 34/1611 (2.11%) |  |  |  | nonsynonymous SNV | SOX9:NM_000346:c.1043A>C:NP_000337:p.Q348P |
| F712070229 | Lung | chr22 | 29090054 | 29090054 | G | A | chr22:g.29090054G>A (NC_000022.10) | 159/2296 (6.93%) | 170/4252 (4.00%) | 0.001 | Conflicting_interpretations_of_pathogenicity | rs142763740 | nonsynonymous SNV | CHEK2:NM_001005735:c.1556C>T:NP_001005735:p.T519M\|CHEK2:NM_001257387:c.764C>T:NP_001244316:p.T255M\|CHEK2:NM_007194:c.1427C>T:NP_009125:p.T476M\|CHEK2:NM_145862:c.1340C>T:NP_665861:p.T447M |
| F712070229 | Lung | chr7 | 6043613 | 6043613 | G | A | chr7:g.6043613G>A (NC_000007.13) | 64/736 (8.70%) | 98/4382 (2.24%) | 0.0011 | Conflicting_interpretations_of_pathogenicity | rs143162541 | stopgain | PMS2:NM_001322007:c.25C>T:NP_001308936:p.R9X\|PMS2:NM_001322008:c.25C>T:NP_001308937:p.R9X |
| F712070229 | Lung | chr7 | 6043666 | 6043666 | C | T | chr7:g.6043666C>T (NC_000007.13) | 43/1225 (3.51%) | 134/6266 (2.14%) | 0.0001 |  | rs772216832 | nonsynonymous SNV | PMS2:NM_000535:c.187G>A:NP_000526:p.V63M\|PMS2:NM_001322006:c.187G>A:NP_001308935:p.V63M\|PMS2:NM_001322014:c.187G>A:NP_001308943:p.V63M |
| F712210236 | Colorectal | chr22 | 29090054 | 29090054 | G | A | chr22:g.29090054G>A (NC_000022.10) | 42/632 (6.65%) | 15/1378 (1.09%) | 0.001 | Conflicting_interpretations_of_pathogenicity | rs142763740 | nonsynonymous SNV | CHEK2:NM_001005735:c.1556C>T:NP_001005735:p.T519M\|CHEK2:NM_001257387:c.764C>T:NP_001244316:p.T255M\|CHEK2:NM_007194:c.1427C>T:NP_009125:p.T476M\|CHEK2:NM_145862:c.1340C>T:NP_665861:p.T447M |
| F712210236 | Colorectal | chr7 | 6043666 | 6043666 | C | T | chr7:g.6043666C>T (NC_000007.13) | 41/533 (7.69%) | 78/1489 (5.24%) | 0.0001 |  | rs772216832 | nonsynonymous SNV | PMS2:NM_000535:c.187G>A:NP_000526:p.V63M\|PMS2:NM_001322006:c.187G>A:NP_001308935:p.V63M\|PMS2:NM_001322014:c.187G>A:NP_001308943:p.V63M |
| F801020001 | Colorectal | chr12 | 49445038 | 49445038 | T | A | chr12:g.49445038T>A (NC_000012.11) | 14/830 (1.69%) | 23/1520 (1.51%) |  |  |  | nonsynonymous SNV | KMT2D:NM_003482:c.2428A>T:NP_003473:p.T810S |
| F801020001 | Colorectal | chr17 | 29527569 | 29527569 | T | G | chr17:g.29527569T>G (NC_000017.10) | 14/726 (1.93%) | 42/2877 (1.46%) |  |  |  | nonsynonymous SNV | NF1:NM_000267:c.1018T>G:NP_000258:p.S340A\|NF1:NM_001042492:c.1018T>G:NP_001035957:p.S340A\|NF1:NM_001128147:c.1018T>G:NP_001121619:p.S340A |
| F801020001 | Colorectal | chr17 | 29541476 | 29541476 | C | T | chr17:g.29541476C>T (NC_000017.10) | 32/536 (5.97%) | 64/2053 (3.12%) |  |  |  | nonsynonymous SNV | NF1:NM_000267:c.1400C>T:NP_000258:p.T467I\|NF1:NM_001042492:c.1400C>T:NP_001035957:p.T467I\|NF1:NM_001128147:c.1400C>T:NP_001121619:p.T467I |
| F801020001 | Colorectal | chr22 | 23654017 | 23654017 | G | A | chr22:g.23654017G>A (NC_000022.10) | 7/603 (1.16%) | 43/1692 (2.54%) |  | Uncertain_significance | rs879255379 | nonsynonymous SNV | BCR:NM_004327:c.3316G>A:NP_004318:p.D1106N\|BCR:NM_021574:c.3184G>A:NP_067585:p.D1062N |
| F801020001 | Colorectal | chr22 | 29090054 | 29090054 | G | A | chr22:g.29090054G>A (NC_000022.10) | 66/954 (6.92%) | 155/2464 (6.29%) | 0.001 | Conflicting_interpretations_of_pathogenicity | rs142763740 | nonsynonymous SNV | CHEK2:NM_001005735:c.1556C>T:NP_001005735:p.T519M\|CHEK2:NM_001257387:c.764C>T:NP_001244316:p.T255M\|CHEK2:NM_007194:c.1427C>T:NP_009125:p.T476M\|CHEK2:NM_145862:c.1340C>T:NP_665861:p.T447M |
| F801020001 | Colorectal | chr3 | 10107113 | 10107113 | G | A | chr3:g.10107113G>A (NC_000003.11) | 25/502 (4.98%) | 39/1738 (2.24%) | 0.0002 |  | rs755975980 | nonsynonymous SNV | FANCD2:NM_001018115:c.2204G>A:NP_001018125:p.R735Q\|FANCD2:NM_001319984:c.2204G>A:NP_001306913:p.R735Q\|FANCD2:NM_033084:c.2204G>A:NP_149075:p.R735Q |
| F801020001 | Colorectal | chr7 | 6043666 | 6043666 | C | T | chr7:g.6043666C>T (NC_000007.13) | 67/796 (8.42%) | 68/2606 (2.61%) | 0.0001 |  | rs772216832 | nonsynonymous SNV | PMS2:NM_000535:c.187G>A:NP_000526:p.V63M\|PMS2:NM_001322006:c.187G>A:NP_001308935:p.V63M\|PMS2:NM_001322014:c.187G>A:NP_001308943:p.V63M |
| F803290034 | Lung | chr7 | 151970873 | 151970873 | C | G | chr7:g.151970873C>G (NC_000007.13) | 40/1094 (3.66%) | 89/2397 (3.71%) |  |  |  | nonsynonymous SNV | KMT2C:NM_170606:c.929G>C:NP_733751:p.C310S |
| F804040035 | Ovarian | chr14 | 103336645 | 103336645 | A | G | chr14:g.103336645A>G (NC_000014.8) | 21/1920 (1.09%) | 10/413 (2.42%) |  |  |  | nonsynonymous SNV | TRAF3:NM_001199427:c.107A>G:NP_001186356:p.Q36R\|TRAF3:NM_003300:c.107A>G:NP_003291:p.Q36R\|TRAF3:NM_145725:c.107A>G:NP_663777:p.Q36R\|TRAF3:NM_145726:c.107A>G:NP_663778:p.Q36R |
| F804040035 | Ovarian | chr17 | 29686028 | 29686028 | T | G | chr17:g.29686028T>G (NC_000017.10) | 14/813 (1.72%) | 3/255 (1.18%) |  |  |  | nonsynonymous SNV | NF1:NM_000267:c.8092T>G:NP_000258:p.S2698A\|NF1:NM_001042492:c.8155T>G:NP_001035957:p.S2719A |
| F804040035 | Ovarian | chr3 | 52588743 | 52588743 | G | T | chr3:g.52588743G>T (NC_000003.11) | 13/1029 (1.26%) | 3/236 (1.27%) |  |  |  | nonsynonymous SNV | PBRM1:NM_018313:c.4285C>A:NP_060783:p.P1429T |
| F804040035 | Ovarian | chr3 | 187451459 | 187451459 | C | A | chr3:g.187451459C>A (NC_000003.11) | 8/584 (1.37%) | 4/169 (2.37%) |  |  |  | nonsynonymous SNV | BCL6:NM_001130845:c.23G>T:NP_001124317:p.C8F\|BCL6:NM_001134738:c.23G>T:NP_001128210:p.C8F\|BCL6:NM_001706:c.23G>T:NP_001697:p.C8F |
| F804040035 | Ovarian | chr7 | 151970889 | 151970889 | T | C | chr7:g.151970889T>C (NC_000007.13) | 64/2910 (2.20%) | 67/1439 (4.66%) |  |  |  | nonsynonymous SNV | KMT2C:NM_170606:c.913A>G:NP_733751:p.M305V |
| F901130009 | Lung | chr17 | 30264515 | 30264515 | G | A | chr17:g.30264515G>A (NC_000017.10) | 54/1199 (4.31%) | 26/1208 (2.11%) |  |  | rs933237275 | nonsynonymous SNV | SUZ12:NM_001321207:c.250G>A:NP_001308136:p.E84K\|SUZ12:NM_015355:c.250G>A:NP_056170:p.E84K |
| F902280085 | Lung | chr20 | 31022477 | 31022478 | CA | - | chr20:g.31022476C>-CA (NC_000020.10) | 29/909 (3.19%) | 6/395 (1.52%) |  |  |  | frameshift deletion | ASXL1:NM_015338:c.1962_1963del:NP_056153:p.A654fs |
| F902280085 | Lung | chr7 | 151841818 | 151841818 | G | C | chr7:g.151841818G>C (NC_000007.13) | 60/622 (9.65%) | 17/304 (5.59%) |  |  |  | nonsynonymous SNV | KMT2C:NM_170606:c.14323C>G:NP_733751:p.L4775V |
| F905150122 | Lung | chr7 | 151945060 | 151945060 | G | A | chr7:g.151945060G>A (NC_000007.13) | 1438/10860 (13.24%) | 704/6717 (10.48%) | 0.001 |  | rs200598064 | nonsynonymous SNV | KMT2C:NM_170606:c.2459C>T:NP_733751:p.T820I |
| F907140148 | Lung | chr19 | 15272519 | 15272519 | T | G | chr19:g.15272519T>G (NC_000019.9) | 13/1130 (1.15%) | 4/152 (2.63%) |  |  |  | nonsynonymous SNV | NOTCH3:NM_000435:c.5920A>C:NP_000426:p.T1974P |
| F907270169 | Hepatobiliary | chr1 | 40366838 | 40366838 | A | T | chr1:g.40366838A>T (NC_000001.10) | 14/595 (2.35%) | 15/598 (2.51%) |  |  |  | nonsynonymous SNV | MYCL:NM_001033081:c.269T>A:NP_001028253:p.I90K\|MYCL:NM_001033082:c.359T>A:NP_001028254:p.I120K\|MYCL:NM_005376:c.359T>A:NP_005367:p.I120K |
| F907270169 | Hepatobiliary | chr22 | 29090061 | 29090061 | G | A | chr22:g.29090061G>A (NC_000022.10) | 16/877 (1.82%) | 17/735 (2.31%) | 0.001 | Conflicting_interpretations_of_pathogenicity | rs540635787 | nonsynonymous SNV | CHEK2:NM_001005735:c.1549C>T:NP_001005735:p.R517C\|CHEK2:NM_001257387:c.757C>T:NP_001244316:p.R253C\|CHEK2:NM_007194:c.1420C>T:NP_009125:p.R474C\|CHEK2:NM_145862:c.1333C>T:NP_665861:p.R445C |
| F909250242 | Ovarian | chr2 | 25457242 | 25457242 | C | T | chr2:g.25457242C>T (NC_000002.11) | 15/1268 (1.18%) | 10/743 (1.35%) | 0.0009 | Conflicting_interpretations_of_pathogenicity | rs147001633 | nonsynonymous SNV | DNMT3A:NM_001320893:c.2189G>A:NP_001307822:p.R730H\|DNMT3A:NM_022552:c.2645G>A:NP_072046:p.R882H\|DNMT3A:NM_153759:c.2078G>A:NP_715640:p.R693H\|DNMT3A:NM_175629:c.2645G>A:NP_783328:p.R882H |
| F910310285 | Lung | chr20 | 31022511 | 31022515 | GGTGA | - | chr20:g.31022510T>-GGTGA (NC_000020.10) | 17/1300 (1.31%) | 4/207 (1.93%) |  |  |  | frameshift deletion | ASXL1:NM_015338:c.1996_2000del:NP_056153:p.G666fs |
| F912080320 | Lung | chrX | 76776310 | 76776310 | G | A | chrX:g.76776310G>A (NC_000023.10) | 28/799 (3.50%) | 13/260 (5.00%) |  | Pathogenic | rs122445099 | stopgain | ATRX:NM_000489:c.7156C>T:NP_000480:p.R2386X\|ATRX:NM_138270:c.7042C>T:NP_612114:p.R2348X |
| F912080321 | Lung | chr17 | 29497001 | 29497001 | A | C | chr17:g.29497001A>C (NC_000017.10) | 8/596 (1.34%) | 6/141 (4.26%) |  | Uncertain_significance | | nonsynonymous SNV | NF1:NM_000267:c.572A>C:NP_000258:p.K191T\|NF1:NM_001042492:c.572A>C:NP_001035957:p.K191T\|NF1:NM_001128147:c.572A>C:NP_001121619:p.K191T |
| F912150333 | Ovarian | chr7 | 151970873 | 151970873 | C | G | chr7:g.151970873C>G (NC_000007.13) | 155/4178 (3.71%) | 26/721 (3.61%) |  |  |  | nonsynonymous SNV | KMT2C:NM_170606:c.929G>C:NP_733751:p.C310S |
| F912190340 | Ovarian | chr17 | 58734167 | 58734167 | A | - | chr17:g.58734166G>-A (NC_000017.10) | 16/770 (2.08%) | 6/209 (2.87%) |  |  |  | stopgain | PPM1D:NM_003620:c.1225delA:NP_003611:p.M409X |
| F912310356 | Endometrial | chr17 | 29497001 | 29497001 | A | C | chr17:g.29497001A>C (NC_000017.10) | 22/1428 (1.54%) | 6/282 (2.13%) |  | Uncertain_significance | | nonsynonymous SNV | NF1:NM_000267:c.572A>C:NP_000258:p.K191T\|NF1:NM_001042492:c.572A>C:NP_001035957:p.K191T\|NF1:NM_001128147:c.572A>C:NP_001121619:p.K191T |
| F912310356 | Endometrial | chr17 | 30267300 | 30267300 | C | T | chr17:g.30267300C>T (NC_000017.10) | 66/669 (9.87%) | 13/121 (10.74%) |  |  | rs1011664623 | intronic | SUZ12 |
| F001190023 | Ovarian | chr7 | 151945109 | 151945109 | G | C | chr7:g.151945109G>C (NC_000007.13) | 214/1943 (11.01%) | 56/1060 (5.28%) | 0.0028 |  | rs199936547 | nonsynonymous SNV | KMT2C:NM_170606:c.2410C>G:NP_733751:p.L804V |
| F002040042 | Colorectal | chr19 | 15291800 | 15291800 | A | C | chr19:g.15291800A>C (NC_000019.9) | 11/668 (1.65%) | 11/604 (1.82%) |  |  |  | nonsynonymous SNV | NOTCH3:NM_000435:c.2966T>G:NP_000426:p.L989R |
| F002080045 | Lung | chr19 | 15291800 | 15291800 | A | C | chr19:g.15291800A>C (NC_000019.9) | 8/516 (1.55%) | 11/755 (1.46%) |  |  |  | nonsynonymous SNV | NOTCH3:NM_000435:c.2966T>G:NP_000426:p.L989R |
| F002080045 | Lung | chr22 | 29092899 | 29092899 | C | T | chr22:g.29092899C>T (NC_000022.10) | 8/536 (1.49%) | 18/1591 (1.13%) | 0.0001 | Uncertain_significance | rs767306337 | nonsynonymous SNV | CHEK2:NM_001005735:c.1214G>A:NP_001005735:p.C405Y\|CHEK2:NM_001257387:c.422G>A:NP_001244316:p.C141Y\|CHEK2:NM_007194:c.1085G>A:NP_009125:p.C362Y |
| F002260070 | Colorectal | chr5 | 1294658 | 1294658 | A | C | chr5:g.1294658A>C (NC_000005.9) | 10/544 (1.84%) | 7/169 (4.14%) |  |  |  | nonsynonymous SNV | TERT:NM_001193376:c.343T>G:NP_001180305:p.F115V\|TERT:NM_198253:c.343T>G:NP_937983:p.F115V |
| F003050077 | Colorectal | chr1 | 27023094 | 27023094 | A | C | chr1:g.27023094A>C (NC_000001.10) | 17/817 (2.08%) | 3/142 (2.11%) |  |  |  | nonsynonymous SNV | ARID1A:NM_006015:c.200A>C:NP_006006:p.Q67P\|ARID1A:NM_139135:c.200A>C:NP_624361:p.Q67P |
| F003050077 | Colorectal | chr1 | 27023100 | 27023100 | T | G | chr1:g.27023100T>G (NC_000001.10) | 8/785 (1.02%) | 3/140 (2.14%) |  |  |  | nonsynonymous SNV | ARID1A:NM_006015:c.206T>G:NP_006006:p.L69R\|ARID1A:NM_139135:c.206T>G:NP_624361:p.L69R |
| F003050077 | Colorectal | chr1 | 27023109 | 27023109 | A | G | chr1:g.27023109A>G (NC_000001.10) | 14/715 (1.96%) | 3/110 (2.73%) |  |  |  | nonsynonymous SNV | ARID1A:NM_006015:c.215A>G:NP_006006:p.E72G\|ARID1A:NM_139135:c.215A>G:NP_624361:p.E72G |
| F003050077 | Colorectal | chr1 | 27023134 | 27023134 | T | G | chr1:g.27023134T>G (NC_000001.10) | 11/1018 (1.08%) | 4/159 (2.52%) |  |  |  | nonsynonymous SNV | ARID1A:NM_006015:c.240T>G:NP_006006:p.N80K\|ARID1A:NM_139135:c.240T>G:NP_624361:p.N80K |
| F003050077 | Colorectal | chr1 | 40366566 | 40366566 | A | C | chr1:g.40366566A>C (NC_000001.10) | 56/4286 (1.31%) | 4/243 (1.65%) |  |  |  | nonsynonymous SNV | MYCL:NM_005376:c.631T>G:NP_005367:p.W211G |
| F003050077 | Colorectal | chr1 | 47691542 | 47691542 | T | G | chr1:g.47691542T>G (NC_000001.10) | 42/1157 (3.63%) | 9/114 (7.89%) |  |  |  | nonsynonymous SNV | TAL1:NM_001287347:c.19A>C:NP_001274276:p.S7R\|TAL1:NM_001290403:c.19A>C:NP_001277332:p.S7R\|TAL1:NM_001290404:c.19A>C:NP_001277333:p.S7R\|TAL1:NM_001290405:c.19A>C:NP_001277334:p.S7R\|TAL1:NM_003189:c.19A>C:NP_003180:p.S7R |
| F003050077 | Colorectal | chr1 | 150550750 | 150550750 | T | G | chr1:g.150550750T>G (NC_000001.10) | 31/2890 (1.07%) | 21/421 (4.99%) |  |  |  | nonsynonymous SNV | MCL1:NM_001197320:c.447A>C:NP_001184249:p.K149N\|MCL1:NM_021960:c.906A>C:NP_068779:p.K302N |
| F003050077 | Colorectal | chr1 | 150551805 | 150551805 | T | G | chr1:g.150551805T>G (NC_000001.10) | 9/615 (1.46%) | 5/256 (1.95%) |  |  |  | nonsynonymous SNV | MCL1:NM_021960:c.202A>C:NP_068779:p.T68P\|MCL1:NM_182763:c.202A>C:NP_877495:p.T68P |
| F003050077 | Colorectal | chr1 | 156843433 | 156843433 | A | C | chr1:g.156843433A>C (NC_000001.10) | 21/1465 (1.43%) | 8/115 (6.96%) |  |  |  | nonsynonymous SNV | NTRK1:NM_001007792:c.769A>C:NP_001007793:p.S257R\|NTRK1:NM_001012331:c.859A>C:NP_001012331:p.S287R\|NTRK1:NM_002529:c.859A>C:NP_002520:p.S287R |
| F003050077 | Colorectal | chr11 | 32456536 | 32456536 | A | C | chr11:g.32456536A>C (NC_000011.9) | 27/1433 (1.88%) | 8/135 (5.93%) |  |  |  | nonsynonymous SNV | WT1:NM_000378:c.356T>G:NP_000369:p.L119W\|WT1:NM_024424:c.356T>G:NP_077742:p.L119W\|WT1:NM_024426:c.356T>G:NP_077744:p.L119W |
| F003050077 | Colorectal | chr11 | 118307354 | 118307354 | G | C | chr11:g.118307354G>C (NC_000011.9) | 23/815 (2.82%) | 12/147 (8.16%) |  |  | rs1275159840 | nonsynonymous SNV | KMT2A:NM_001197104:c.127G>C:NP_001184033:p.G43R\|KMT2A:NM_005933:c.127G>C:NP_005924:p.G43R |
| F003050077 | Colorectal | chr11 | 118307406 | 118307406 | T | G | chr11:g.118307406T>G (NC_000011.9) | 26/939 (2.77%) | 9/154 (5.84%) |  |  |  | nonsynonymous SNV | KMT2A:NM_001197104:c.179T>G:NP_001184033:p.V60G\|KMT2A:NM_005933:c.179T>G:NP_005924:p.V60G |
| F003050077 | Colorectal | chr11 | 118307624 | 118307624 | T | G | chr11:g.118307624T>G (NC_000011.9) | 95/868 (10.94%) | 24/105 (22.86%) |  |  |  | nonsynonymous SNV | KMT2A:NM_001197104:c.397T>G:NP_001184033:p.F133V\|KMT2A:NM_005933:c.397T>G:NP_005924:p.F133V |
| F003050077 | Colorectal | chr13 | 32912387 | 32912387 | G | T | chr13:g.32912387G>T (NC_000013.10) | 15/1204 (1.25%) | 16/792 (2.02%) |  |  |  | stopgain | BRCA2:NM_000059:c.3895G>T:NP_000050:p.E1299X |
| F003050077 | Colorectal | chr13 | 48878061 | 48878061 | A | C | chr13:g.48878061A>C (NC_000013.10) | 12/774 (1.55%) | 8/198 (4.04%) |  | Uncertain_significance | rs898303682 | nonsynonymous SNV | RB1:NM_000321:c.13A>C:NP_000312:p.T5P |
| F003050077 | Colorectal | chr13 | 48878068 | 48878068 | G | C | chr13:g.48878068G>C (NC_000013.10) | 14/1140 (1.23%) | 7/283 (2.47%) |  |  |  | nonsynonymous SNV | RB1:NM_000321:c.20G>C:NP_000312:p.R7P |
| F003050077 | Colorectal | chr13 | 48878082 | 48878082 | A | C | chr13:g.48878082A>C (NC_000013.10) | 42/1240 (3.39%) | 26/311 (8.36%) |  |  |  | nonsynonymous SNV | RB1:NM_000321:c.34A>C:NP_000312:p.T12P |
| F003050077 | Colorectal | chr14 | 36986875 | 36986875 | T | G | chr14:g.36986875T>G (NC_000014.8) | 29/1401 (2.07%) | 10/218 (4.59%) |  |  |  | nonsynonymous SNV | NKX2-1:NM_001079668:c.814A>C:NP_001073136:p.T272P\|NKX2-1:NM_003317:c.724A>C:NP_003308:p.T242P |
| F003050077 | Colorectal | chr14 | 104169601 | 104169601 | T | G | chr14:g.104169601T>G (NC_000014.8) | 51/885 (5.76%) | 22/227 (9.69%) |  |  |  | nonsynonymous SNV | XRCC3:NM_001100118:c.470A>C:NP_001093588:p.Q157P\|XRCC3:NM_001100119:c.470A>C:NP_001093589:p.Q157P\|XRCC3:NM_005432:c.470A>C:NP_005423:p.Q157P |
| F003050077 | Colorectal | chr15 | 33010283 | 33010283 | A | C | chr15:g.33010283A>C (NC_000015.9) | 10/770 (1.30%) | 7/131 (5.34%) |  |  |  | UTR5 | GREM1(NM_013372:c.-12609A>C,NM_001191323:c.-12609A>C,NM_001191322:c.-12609A>C) |
| F003050077 | Colorectal | chr17 | 58677984 | 58677984 | T | G | chr17:g.58677984T>G (NC_000017.10) | 13/962 (1.35%) | 3/174 (1.72%) |  |  |  | nonsynonymous SNV | PPM1D:NM_003620:c.209T>G:NP_003611:p.V70G |
| F003050077 | Colorectal | chr17 | 70119725 | 70119725 | A | C | chr17:g.70119725A>C (NC_000017.10) | 28/871 (3.21%) | 4/112 (3.57%) |  |  |  | nonsynonymous SNV | SOX9:NM_000346:c.727A>C:NP_000337:p.T243P |
| F003050077 | Colorectal | chr17 | 70120038 | 70120038 | A | C | chr17:g.70120038A>C (NC_000017.10) | 26/1545 (1.68%) | 10/228 (4.39%) |  |  |  | nonsynonymous SNV | SOX9:NM_000346:c.1040A>C:NP_000337:p.Q347P |
| F003050077 | Colorectal | chr17 | 70120041 | 70120041 | A | C | chr17:g.70120041A>C (NC_000017.10) | 17/1385 (1.23%) | 3/214 (1.40%) |  |  |  | nonsynonymous SNV | SOX9:NM_000346:c.1043A>C:NP_000337:p.Q348P |
| F003050077 | Colorectal | chr18 | 60985734 | 60985734 | T | G | chr18:g.60985734T>G (NC_000018.9) | 26/2202 (1.18%) | 5/362 (1.38%) |  |  |  | nonsynonymous SNV | BCL2:NM_000633:c.166A>C:NP_000624:p.T56P\|BCL2:NM_000657:c.166A>C:NP_000648:p.T56P |
| F003050077 | Colorectal | chr19 | 2226859 | 2226879 | GCGGCGTCCTCCGCAGGCGGC | - | chr19:g.2226858G>-GCGGCGTCCTCCGCAGGCGGC (NC_000019.9) | 164/2367 (6.93%) | 20/291 (6.87%) |  |  | rs748637415 | nonframeshift deletion | DOT1L:NM_032482:c.4339_4359del:NP_115871:p.1447_1453del |
| F003050077 | Colorectal | chr19 | 15272329 | 15272329 | T | G | chr19:g.15272329T>G (NC_000019.9) | 48/2807 (1.71%) | 7/272 (2.57%) |  |  |  | nonsynonymous SNV | NOTCH3:NM_000435:c.6110A>C:NP_000426:p.H2037P |
| F003050077 | Colorectal | chr19 | 15290906 | 15290906 | A | C | chr19:g.15290906A>C (NC_000019.9) | 21/1896 (1.11%) | 8/248 (3.23%) |  |  |  | nonsynonymous SNV | NOTCH3:NM_000435:c.3304T>G:NP_000426:p.Y1102D |
| F003050077 | Colorectal | chr19 | 15292552 | 15292552 | A | C | chr19:g.15292552A>C (NC_000019.9) | 28/893 (3.14%) | 10/215 (4.65%) |  |  |  | nonsynonymous SNV | NOTCH3:NM_000435:c.2627T>G:NP_000426:p.L876R |
| F003050077 | Colorectal | chr19 | 33792554 | 33792554 | A | C | chr19:g.33792554A>C (NC_000019.9) | 37/2275 (1.63%) | 6/281 (2.14%) |  |  |  | nonsynonymous SNV | CEBPA:NM_001285829:c.410T>G:NP_001272758:p.L137R\|CEBPA:NM_001287424:c.872T>G:NP_001274353:p.L291R\|CEBPA:NM_001287435:c.725T>G:NP_001274364:p.L242R\|CEBPA:NM_004364:c.767T>G:NP_004355:p.L256R |
| F003050077 | Colorectal | chr19 | 33792947 | 33792947 | T | G | chr19:g.33792947T>G (NC_000019.9) | 17/545 (3.12%) | 5/129 (3.88%) |  |  |  | nonsynonymous SNV | CEBPA:NM_001285829:c.17A>C:NP_001272758:p.H6P\|CEBPA:NM_001287424:c.479A>C:NP_001274353:p.H160P\|CEBPA:NM_001287435:c.332A>C:NP_001274364:p.H111P\|CEBPA:NM_004364:c.374A>C:NP_004355:p.H125P |
| F003050077 | Colorectal | chr19 | 33792965 | 33792965 | A | C | chr19:g.33792965A>C (NC_000019.9) | 24/845 (2.84%) | 5/128 (3.91%) |  |  |  | nonsynonymous SNV | CEBPA:NM_001287424:c.461T>G:NP_001274353:p.V154G\|CEBPA:NM_001287435:c.314T>G:NP_001274364:p.V105G\|CEBPA:NM_004364:c.356T>G:NP_004355:p.V119G |
| F003050077 | Colorectal | chr19 | 33792975 | 33792975 | C | G | chr19:g.33792975C>G (NC_000019.9) | 40/1367 (2.93%) | 4/161 (2.48%) |  |  |  | nonsynonymous SNV | CEBPA:NM_001287424:c.451G>C:NP_001274353:p.G151R\|CEBPA:NM_001287435:c.304G>C:NP_001274364:p.G102R\|CEBPA:NM_004364:c.346G>C:NP_004355:p.G116R |
| F003050077 | Colorectal | chr19 | 33793250 | 33793250 | T | G | chr19:g.33793250T>G (NC_000019.9) | 17/1613 (1.05%) | 4/260 (1.54%) |  |  |  | nonsynonymous SNV | CEBPA:NM_001287424:c.176A>C:NP_001274353:p.H59P\|CEBPA:NM_001287435:c.29A>C:NP_001274364:p.H10P\|CEBPA:NM_004364:c.71A>C:NP_004355:p.H24P |
| F003050077 | Colorectal | chr19 | 36209221 | 36209221 | T | G | chr19:g.36209221T>G (NC_000019.9) | 46/781 (5.89%) | 11/102 (10.78%) |  |  |  | nonsynonymous SNV | KMT2B:NM_014727:c.301T>G:NP_055542:p.W101G |
| F003050077 | Colorectal | chr2 | 16082652 | 16082652 | A | C | chr2:g.16082652A>C (NC_000002.11) | 28/1326 (2.11%) | 5/157 (3.18%) |  |  |  | nonsynonymous SNV | MYCN:NM_001293228:c.466A>C:NP_001280157:p.S156R\|MYCN:NM_005378:c.466A>C:NP_005369:p.S156R |
| F003050077 | Colorectal | chr22 | 23523455 | 23523455 | A | C | chr22:g.23523455A>C (NC_000022.10) | 34/1856 (1.83%) | 6/253 (2.37%) |  |  |  | nonsynonymous SNV | BCR:NM_004327:c.308A>C:NP_004318:p.D103A\|BCR:NM_021574:c.308A>C:NP_067585:p.D103A |
| F003050077 | Colorectal | chr3 | 10183674 | 10183674 | T | G | chr3:g.10183674T>G (NC_000003.11) | 10/991 (1.01%) | 9/314 (2.87%) | 0.0001 | Uncertain_significance | rs199959170 | nonsynonymous SNV | VHL:NM_000551:c.143T>G:NP_000542:p.L48R\|VHL:NM_198156:c.143T>G:NP_937799:p.L48R |
| F003050077 | Colorectal | chr3 | 128204657 | 128204657 | T | G | chr3:g.128204657T>G (NC_000003.11) | 32/1926 (1.66%) | 5/191 (2.62%) |  |  |  | nonsynonymous SNV | GATA2:NM_001145661:c.784A>C:NP_001139133:p.S262R\|GATA2:NM_001145662:c.784A>C:NP_001139134:p.S262R\|GATA2:NM_032638:c.784A>C:NP_116027:p.S262R |
| F003050077 | Colorectal | chr3 | 181430993 | 181430993 | A | C | chr3:g.181430993A>C (NC_000003.11) | 14/1050 (1.33%) | 5/240 (2.08%) |  |  |  | nonsynonymous SNV | SOX2:NM_003106:c.845A>C:NP_003097:p.E282A |
| F003050077 | Colorectal | chr5 | 1294884 | 1294884 | A | C | chr5:g.1294884A>C (NC_000005.9) | 32/3173 (1.01%) | 3/165 (1.82%) |  |  |  | splicing | TERT(NM_001193376:exon1:c.219+2T>G,NM_198253:exon1:c.219+2T>G) |
| F003050077 | Colorectal | chr5 | 56111503 | 56111503 | A | C | chr5:g.56111503A>C (NC_000005.9) | 41/621 (6.60%) | 20/199 (10.05%) |  |  |  | nonsynonymous SNV | MAP3K1:NM_005921:c.103A>C:NP_005912:p.S35R |
| F003050077 | Colorectal | chr5 | 176520277 | 176520277 | A | C | chr5:g.176520277A>C (NC_000005.9) | 17/1065 (1.60%) | 3/169 (1.78%) |  |  | rs879104864 | nonsynonymous SNV | FGFR4:NM_002011:c.1196A>C:NP_002002:p.H399P\|FGFR4:NM_213647:c.1196A>C:NP_998812:p.H399P |
| F003050077 | Colorectal | chr6 | 35420377 | 35420377 | T | G | chr6:g.35420377T>G (NC_000006.11) | 15/1187 (1.26%) | 3/240 (1.25%) |  |  |  | nonsynonymous SNV | FANCE:NM_021922:c.55T>G:NP_068741:p.W19G |
| F003050077 | Colorectal | chr6 | 157099100 | 157099100 | A | C | chr6:g.157099100A>C (NC_000006.11) | 8/721 (1.11%) | 7/111 (6.31%) |  |  |  | nonsynonymous SNV | ARID1B:NM_017519:c.37A>C:NP_059989:p.T13P\|ARID1B:NM_020732:c.37A>C:NP_065783:p.T13P |
| F003050077 | Colorectal | chr6 | 157099578 | 157099578 | A | C | chr6:g.157099578A>C (NC_000006.11) | 10/713 (1.40%) | 6/140 (4.29%) |  |  | rs914813402 | nonsynonymous SNV | ARID1B:NM_017519:c.515A>C:NP_059989:p.D172A\|ARID1B:NM_020732:c.515A>C:NP_065783:p.D172A |
| F003050077 | Colorectal | chr6 | 157100252 | 157100252 | T | G | chr6:g.157100252T>G (NC_000006.11) | 53/996 (5.32%) | 9/133 (6.77%) |  |  |  | nonsynonymous SNV | ARID1B:NM_017519:c.1189T>G:NP_059989:p.S397A\|ARID1B:NM_020732:c.1189T>G:NP_065783:p.S397A |
| F003050077 | Colorectal | chr7 | 101460757 | 101460757 | T | G | chr7:g.101460757T>G (NC_000007.13) | 32/748 (4.28%) | 7/110 (6.36%) |  |  |  | intronic | CUX1 |
| F003050077 | Colorectal | chr7 | 101460845 | 101460845 | A | C | chr7:g.101460845A>C (NC_000007.13) | 12/867 (1.38%) | 8/139 (5.76%) |  |  |  | intronic | CUX1 |
| F003050077 | Colorectal | chr7 | 101460865 | 101460865 | A | C | chr7:g.101460865A>C (NC_000007.13) | 18/1637 (1.10%) | 5/189 (2.65%) |  |  |  | intronic | CUX1 |
| F003050077 | Colorectal | chr7 | 101891942 | 101891942 | A | C | chr7:g.101891942A>C (NC_000007.13) | 59/2263 (2.61%) | 7/168 (4.17%) |  |  |  | nonsynonymous SNV | CUX1:NM_001202543:c.4171A>C:NP_001189472:p.T1391P\|CUX1:NM_181552:c.4138A>C:NP_853530:p.T1380P |
| F003050077 | Colorectal | chr7 | 101891955 | 101891955 | A | C | chr7:g.101891955A>C (NC_000007.13) | 33/2288 (1.44%) | 4/174 (2.30%) |  |  |  | nonsynonymous SNV | CUX1:NM_001202543:c.4184A>C:NP_001189472:p.D1395A\|CUX1:NM_181552:c.4151A>C:NP_853530:p.D1384A |
| F003050077 | Colorectal | chr7 | 101892029 | 101892029 | A | C | chr7:g.101892029A>C (NC_000007.13) | 27/1699 (1.59%) | 3/199 (1.51%) |  |  |  | nonsynonymous SNV | CUX1:NM_001202543:c.4258A>C:NP_001189472:p.T1420P\|CUX1:NM_181552:c.4225A>C:NP_853530:p.T1409P |
| F003050077 | Colorectal | chr7 | 128829074 | 128829074 | C | G | chr7:g.128829074C>G (NC_000007.13) | 9/752 (1.20%) | 4/107 (3.74%) |  |  |  | nonsynonymous SNV | SMO:NM_005631:c.82C>G:NP_005622:p.R28G |
| F003050077 | Colorectal | chr7 | 152132790 | 152132790 | T | G | chr7:g.152132790T>G (NC_000007.13) | 13/811 (1.60%) | 6/141 (4.26%) |  |  | rs1309606619 | nonsynonymous SNV | KMT2C:NM_170606:c.82A>C:NP_733751:p.S28R |
| F003050077 | Colorectal | chr8 | 128751215 | 128751215 | A | C | chr8:g.128751215A>C (NC_000008.10) | 41/1609 (2.55%) | 24/293 (8.19%) |  |  |  | nonsynonymous SNV | MYC:NM_002467:c.752A>C:NP_002458:p.E251A |
| F003050077 | Colorectal | chr9 | 139399928 | 139399928 | A | C | chr9:g.139399928A>C (NC_000009.11) | 17/1648 (1.03%) | 4/272 (1.47%) |  |  |  | nonsynonymous SNV | NOTCH1:NM_017617:c.4420T>G:NP_060087:p.W1474G |
| F003290111 | Lung | chr20 | 57484427 | 57484427 | T | C | chr20:g.57484427T>C (NC_000020.10) | 10/524 (1.91%) | 3/185 (1.62%) |  |  |  | nonsynonymous SNV | GNAS:NM_000516:c.608T>C:NP_000507:p.L203P\|GNAS:NM_001077488:c.611T>C:NP_001070956:p.L204P\|GNAS:NM_001077489:c.563T>C:NP_001070957:p.L188P\|GNAS:NM_001309840:c.431T>C:NP_001296769:p.L144P\|GNAS:NM_001309861:c.431T>C:NP_001296790:p.L144P\|GNAS:NM_080425:c.2537T>C:NP_536350:p.L846P\|GNAS:NM_080426:c.566T>C:NP_536351:p.L189P |
| F003290111 | Lung | chr6 | 138192623 | 138192623 | C | T | chr6:g.138192623C>T (NC_000006.11) | 38/2093 (1.82%) | 21/540 (3.89%) |  |  |  | stopgain | TNFAIP3:NM_001270507:c.259C>T:NP_001257436:p.R87X\|TNFAIP3:NM_001270508:c.259C>T:NP_001257437:p.R87X\|TNFAIP3:NM_006290:c.259C>T:NP_006281:p.R87X |
| F003290111 | Lung | chr7 | 151970840 | 151970840 | C | T | chr7:g.151970840C>T (NC_000007.13) | 357/2125 (16.80%) | 95/484 (19.63%) | 0.0007 |  | rs780548283 | nonsynonymous SNV | KMT2C:NM_170606:c.962G>A:NP_733751:p.S321N |
| F004060123 | Lung | chr7 | 151970840 | 151970840 | C | T | chr7:g.151970840C>T (NC_000007.13) | 299/2651 (11.28%) | 145/834 (17.39%) | 0.0007 |  | rs780548283 | nonsynonymous SNV | KMT2C:NM_170606:c.962G>A:NP_733751:p.S321N |
| F004060123 | Lung | chr8 | 41789794 | 41789794 | A | T | chr8:g.41789794A>T (NC_000008.10) | 8/764 (1.05%) | 4/293 (1.37%) |  |  |  | nonsynonymous SNV | KAT6A:NM_006766:c.5944T>A:NP_006757:p.S1982T |
| F004160134 | Lung | chr14 | 81557414 | 81557414 | G | C | chr14:g.81557414G>C (NC_000014.8) | 193/1127 (17.13%) | 176/392 (44.90%) | 0.0005 | Benign(1),Likely_pathogenic(1),Uncertain_significance(1) | rs760874290 | nonsynonymous SNV | TSHR:NM_000369:c.394G>C:NP_000360:p.G132R\|TSHR:NM_001018036:c.394G>C:NP_001018046:p.G132R\|TSHR:NM_001142626:c.394G>C:NP_001136098:p.G132R |
| F004210139 | Lung | chr16 | 2120571 | 2120571 | C | T | chr16:g.2120571C>T (NC_000016.9) | 63/568 (11.09%) | 12/198 (6.06%) |  | Pathogenic | rs45469298 | nonsynonymous SNV | TSC2:NM_000548:c.1831C>T:NP_000539:p.R611W\|TSC2:NM_001077183:c.1831C>T:NP_001070651:p.R611W\|TSC2:NM_001114382:c.1831C>T:NP_001107854:p.R611W\|TSC2:NM_001318827:c.1720C>T:NP_001305756:p.R574W\|TSC2:NM_001318829:c.1684C>T:NP_001305758:p.R562W\|TSC2:NM_001318831:c.1231C>T:NP_001305760:p.R411W\|TSC2:NM_001318832:c.1864C>T:NP_001305761:p.R622W |
| F004210140 | Lung | chr5 | 170819954 | 170819956 | GAT | - | chr5:g.170819953C>-GAT (NC_000005.9) | 76/609 (12.48%) | 39/168 (23.21%) | 0.0002 |  | rs751654967 | nonframeshift deletion | NPM1:NM_001037738:c.496_498del:NP_001032827:p.166_166del\|NPM1:NM_002520:c.496_498del:NP_002511:p.166_166del\|NPM1:NM_199185:c.496_498del:NP_954654:p.166_166del |
| F004260142 | Breast | chr2 | 25457242 | 25457242 | C | T | chr2:g.25457242C>T (NC_000002.11) | 140/1110 (12.61%) | 28/278 (10.07%) | 0.0009 | Pathogenic(2),Uncertain_significance(1) | rs147001633 | nonsynonymous SNV | DNMT3A:NM_001320893:c.2189G>A:NP_001307822:p.R730H\|DNMT3A:NM_022552:c.2645G>A:NP_072046:p.R882H\|DNMT3A:NM_153759:c.2078G>A:NP_715640:p.R693H\|DNMT3A:NM_175629:c.2645G>A:NP_783328:p.R882H |
| F004280146 | Lung | chr12 | 49446159 | 49446159 | T | G | chr12:g.49446159T>G (NC_000012.11) | 8/714 (1.12%) | 9/231 (3.90%) |  |  |  | nonsynonymous SNV | KMT2D:NM_003482:c.1307A>C:NP_003473:p.E436A |
| F004280146 | Lung | chr16 | 67671694 | 67671694 | A | C | chr16:g.67671694A>C (NC_000016.9) | 13/649 (2.00%) | 11/704 (1.56%) |  |  |  | nonsynonymous SNV | CTCF:NM_001191022:c.1119A>C:NP_001177951:p.E373D\|CTCF:NM_006565:c.2103A>C:NP_006556:p.E701D |
| F004280146 | Lung | chr17 | 17119697 | 17119697 | A | C | chr17:g.17119697A>C (NC_000017.10) | 34/665 (5.11%) | 18/647 (2.78%) |  |  |  | nonsynonymous SNV | FLCN:NM_144997:c.1297T>G:NP_659434:p.S433A |
| F004280146 | Lung | chr19 | 15291800 | 15291800 | A | C | chr19:g.15291800A>C (NC_000019.9) | 21/1004 (2.09%) | 16/645 (2.48%) |  |  |  | nonsynonymous SNV | NOTCH3:NM_000435:c.2966T>G:NP_000426:p.L989R |
| F004280146 | Lung | chr19 | 15292552 | 15292552 | A | C | chr19:g.15292552A>C (NC_000019.9) | 15/563 (2.66%) | 9/506 (1.78%) |  |  |  | nonsynonymous SNV | NOTCH3:NM_000435:c.2627T>G:NP_000426:p.L876R |
| F004280146 | Lung | chr19 | 18279283 | 18279283 | A | C | chr19:g.18279283A>C (NC_000019.9) | 9/803 (1.12%) | 12/484 (2.48%) |  |  |  | splicing | PIK3R2(NM_005027:exon14:c.1737-2A>C) |
| F004280146 | Lung | chr2 | 25536789 | 25536789 | T | C | chr2:g.25536789T>C (NC_000002.11) | 19/983 (1.93%) | 5/496 (1.01%) |  |  |  | nonsynonymous SNV | DNMT3A:NM_001320892:c.65A>G:NP_001307821:p.D22G\|DNMT3A:NM_022552:c.65A>G:NP_072046:p.D22G\|DNMT3A:NM_175629:c.65A>G:NP_783328:p.D22G\|DNMT3A:NM_175630:c.65A>G:NP_783329:p.D22G |
| F004280146 | Lung | chr22 | 41569690 | 41569690 | A | C | chr22:g.41569690A>C (NC_000022.10) | 7/659 (1.06%) | 12/478 (2.51%) |  |  |  | nonsynonymous SNV | EP300:NM_001429:c.4681A>C:NP_001420:p.S1561R |
| F004280146 | Lung | chr3 | 128204657 | 128204657 | T | G | chr3:g.128204657T>G (NC_000003.11) | 21/692 (3.03%) | 11/427 (2.58%) |  |  |  | nonsynonymous SNV | GATA2:NM_001145661:c.784A>C:NP_001139133:p.S262R\|GATA2:NM_001145662:c.784A>C:NP_001139134:p.S262R\|GATA2:NM_032638:c.784A>C:NP_116027:p.S262R |
| F004280146 | Lung | chr4 | 106157629 | 106157636 | AATAAAGA | - | chr4:g.106157628G>-AATAAAGA (NC_000004.11) | 15/949 (1.58%) | 39/1245 (3.13%) |  |  |  | frameshift deletion | TET2:NM_001127208:c.2530_2537del:NP_001120680:p.N844fs\|TET2:NM_017628:c.2530_2537del:NP_060098:p.N844fs |
| F004280146 | Lung | chr5 | 149440482 | 149440482 | T | G | chr5:g.149440482T>G (NC_000005.9) | 10/953 (1.05%) | 14/472 (2.97%) |  |  |  | nonsynonymous SNV | CSF1R:NM_001288705:c.1912A>C:NP_001275634:p.S638R\|CSF1R:NM_005211:c.1912A>C:NP_005202:p.S638R |
| F004280146 | Lung | chr7 | 101844943 | 101844943 | A | C | chr7:g.101844943A>C (NC_000007.13) | 9/831 (1.08%) | 6/497 (1.21%) |  |  |  | nonsynonymous SNV | CUX1:NM_001202543:c.2399A>C:NP_001189472:p.Q800P\|CUX1:NM_181552:c.2366A>C:NP_853530:p.Q789P |
| F004280146 | Lung | chr9 | 135771732 | 135771732 | C | G | chr9:g.135771732C>G (NC_000009.11) | 9/740 (1.22%) | 10/768 (1.30%) |  |  |  | nonsynonymous SNV | TSC1:NM_000368:c.3385G>C:NP_000359:p.A1129P\|TSC1:NM_001162426:c.3382G>C:NP_001155898:p.A1128P\|TSC1:NM_001162427:c.3232G>C:NP_001155899:p.A1078P |
| F006030203 | Ovarian | chr2 | 25468134 | 25468134 | G | T | chr2:g.25468134G>T (NC_000002.11) | 24/1378 (1.74%) | 4/363 (1.10%) |  |  |  | stopgain | DNMT3A:NM_001320893:c.1086C>A:NP_001307822:p.C362X\|DNMT3A:NM_022552:c.1542C>A:NP_072046:p.C514X\|DNMT3A:NM_153759:c.975C>A:NP_715640:p.C325X\|DNMT3A:NM_175629:c.1542C>A:NP_783328:p.C514X |
| F007050248 | Ovarian | chr4 | 106196458 | 106196458 | C | - | chr4:g.106196457T>-C (NC_000004.11) | 9/882 (1.02%) | 7/566 (1.24%) |  |  |  | frameshift deletion | TET2:NM_001127208:c.4791delC:NP_001120680:p.F1597fs |
| F007010246 | Lung | chr2 | 25467133 | 25467133 | C | T | chr2:g.25467133C>T (NC_000002.11) | 43/1911 (2.25%) | 7/318 (2.20%) |  |  | rs1050697275 | stopgain | DNMT3A:NM_001320893:c.1286G>A:NP_001307822:p.W429X\|DNMT3A:NM_022552:c.1742G>A:NP_072046:p.W581X\|DNMT3A:NM_153759:c.1175G>A:NP_715640:p.W392X\|DNMT3A:NM_175629:c.1742G>A:NP_783328:p.W581X |
| F007010246 | Lung | chr7 | 151970877 | 151970877 | G | C | chr7:g.151970877G>C (NC_000007.13) | 258/5506 (4.69%) | 107/2676 (4.00%) |  |  |  | nonsynonymous SNV | KMT2C:NM_170606:c.925C>G:NP_733751:p.P309A |
| F006180223 | Endometrial | chr17 | 29497001 | 29497001 | A | C | chr17:g.29497001A>C (NC_000017.10) | 9/528 (1.70%) | 11/281 (3.91%) |  | Uncertain_significance | rs1555607113 | nonsynonymous SNV | NF1:NM_000267:c.572A>C:NP_000258:p.K191T\|NF1:NM_001042492:c.572A>C:NP_001035957:p.K191T\|NF1:NM_001128147:c.572A>C:NP_001121619:p.K191T |
| F006090212 | Ovarian | chr3 | 10106517 | 10106517 | C | T | chr3:g.10106517C>T (NC_000003.11) | 41/670 (6.12%) | 6/215 (2.79%) |  |  | rs1256669140 | nonsynonymous SNV | FANCD2:NM_001018115:c.2126C>T:NP_001018125:p.A709V\|FANCD2:NM_001319984:c.2126C>T:NP_001306913:p.A709V\|FANCD2:NM_033084:c.2126C>T:NP_149075:p.A709V |
