# Supplementary material for: Clinical application of liquid biopsy in cancer patients
Source: BMC Cancer. 2022 Apr 15;22:413. doi: 10.1186/s12885-022-09525-0 (PMC9011972; doi:10.1186/s12885-022-09525-0)
Supplement: Supplementary file 3 — Additional file 3: Table S3. Correlation between cancer stage and CH-related variants. [file 12885_2022_9525_MOESM3_ESM.docx]

|  |  | Stage | | | |
| --- | --- | --- | --- | --- | --- |
|  |  | I, II | III, IV | total | p-value |
| CH-related variants | Yes | 2 | 28 | 30 | 0.3058 |
|  | No | 8 | 42 | 50 |  |
| p-value by Fisher's Exact Test | |  |  |  |  |
