# Supplementary material for: Clinical application of liquid biopsy in cancer patients
Source: BMC Cancer. 2022 Apr 15;22:413. doi: 10.1186/s12885-022-09525-0 (PMC9011972; doi:10.1186/s12885-022-09525-0)
Supplement: Supplementary file 4 — Additional file 4: Table S4. cfDNA CH-related variants list in healthy individuals. [file 12885_2022_9525_MOESM4_ESM.docx]

| Sample_ID | Chrom | start_pos | end_pos | ref | alt | Variants | Depth_and_Ratio in plasma | Depth_and_Ratio in WBCs | Allele_Freq | clinvar | avsnp | Type | HGVS |
| --- | --- | --- | --- | --- | --- | --- | --- | --- | --- | --- | --- | --- | --- |
| F711140219 | chr22 | 29090054 | 29090054 | G | A | chr22:g.29090054G>A (NC_000022.10) | 67/601 (11.15%) | 11/225 (4.89%) | 0.001 | Conflicting_interpretations_of_pathogenicity | rs142763740 | nonsynonymous SNV | CHEK2:NM_001005735:c.1556C>T:NP_001005735:p.T519M\|CHEK2:NM_001257387:c.764C>T:NP_001244316:p.T255M\|CHEK2:NM_007194:c.1427C>T:NP_009125:p.T476M\|CHEK2:NM_145862:c.1340C>T:NP_665861:p.T447M |
| F711140220 | chr22 | 29090054 | 29090054 | G | A | chr22:g.29090054G>A (NC_000022.10) | 23/674 (3.41%) | 15/343 (4.37%) | 0.001 | Conflicting_interpretations_of_pathogenicity | rs142763740 | nonsynonymous SNV | CHEK2:NM_001005735:c.1556C>T:NP_001005735:p.T519M\|CHEK2:NM_001257387:c.764C>T:NP_001244316:p.T255M\|CHEK2:NM_007194:c.1427C>T:NP_009125:p.T476M\|CHEK2:NM_145862:c.1340C>T:NP_665861:p.T447M |
| F712210235 | chr16 | 2138243 | 2138243 | C | T | chr16:g.2138243C>T (NC_000016.9) | 17/778 (2.19%) | 19/1241 (1.53%) | 0.0001 | Uncertain_significance | rs761618860 | nonsynonymous SNV | TSC2:NM_000548:c.5176C>T:NP_000539:p.H1726Y\|TSC2:NM_001077183:c.4975C>T:NP_001070651:p.H1659Y\|TSC2:NM_001114382:c.5107C>T:NP_001107854:p.H1703Y\|TSC2:NM_001318827:c.4867C>T:NP_001305756:p.H1623Y\|TSC2:NM_001318829:c.4831C>T:NP_001305758:p.H1611Y\|TSC2:NM_001318831:c.4444C>T:NP_001305760:p.H1482Y\|TSC2:NM_001318832:c.5008C>T:NP_001305761:p.H1670Y |
| F712210235 | chr17 | 29527569 | 29527569 | T | G | chr17:g.29527569T>G (NC_000017.10) | 17/1441 (1.18%) | 54/3178 (1.70%) |  |  |  | nonsynonymous SNV | NF1:NM_000267:c.1018T>G:NP_000258:p.S340A\|NF1:NM_001042492:c.1018T>G:NP_001035957:p.S340A\|NF1:NM_001128147:c.1018T>G:NP_001121619:p.S340A |
| F712210235 | chr17 | 29528142 | 29528142 | T | C | chr17:g.29528142T>C (NC_000017.10) | 33/1109 (2.98%) | 71/3011 (2.36%) |  |  |  | nonsynonymous SNV | NF1:NM_000267:c.1150T>C:NP_000258:p.F384L\|NF1:NM_001042492:c.1150T>C:NP_001035957:p.F384L\|NF1:NM_001128147:c.1150T>C:NP_001121619:p.F384L |
| F712210235 | chr21 | 44513243 | 44513243 | G | A | chr21:g.44513243G>A (NC_000021.8) | 7/657 (1.07%) | 9/743 (1.21%) | 0.0006 |  | rs200044775 | nonsynonymous SNV | U2AF1:NM_001025203:c.692C>T:NP_001020374:p.S231L\|U2AF1:NM_001025204:c.473C>T:NP_001020375:p.S158L\|U2AF1:NM_006758:c.692C>T:NP_006749:p.S231L\|U2AF1L5:NM_001320646:c.692C>T:NP_001307575:p.S231L\|U2AF1L5:NM_001320648:c.692C>T:NP_001307577:p.S231L\|U2AF1L5:NM_001320650:c.578C>T:NP_001307579:p.S193L\|U2AF1L5:NM_001320651:c.473C>T:NP_001307580:p.S158L |
| F712210235 | chr7 | 6043666 | 6043666 | C | T | chr7:g.6043666C>T (NC_000007.13) | 57/1226 (4.65%) | 112/2572 (4.35%) | 0.0001 |  | rs772216832 | nonsynonymous SNV | PMS2:NM_000535:c.187G>A:NP_000526:p.V63M\|PMS2:NM_001322006:c.187G>A:NP_001308935:p.V63M\|PMS2:NM_001322014:c.187G>A:NP_001308943:p.V63M |
| F712260238 | chr22 | 23651650 | 23651650 | A | G | chr22:g.23651650A>G (NC_000022.10) | 10/673 (1.49%) | 10/965 (1.04%) | 0.0002 |  | rs746213513 | nonsynonymous SNV | BCR:NM_004327:c.3052A>G:NP_004318:p.T1018A\|BCR:NM_021574:c.2920A>G:NP_067585:p.T974A |
| F712260238 | chr22 | 29090054 | 29090054 | G | A | chr22:g.29090054G>A (NC_000022.10) | 32/714 (4.48%) | 10/903 (1.11%) | 0.001 | Conflicting_interpretations_of_pathogenicity | rs142763740 | nonsynonymous SNV | CHEK2:NM_001005735:c.1556C>T:NP_001005735:p.T519M\|CHEK2:NM_001257387:c.764C>T:NP_001244316:p.T255M\|CHEK2:NM_007194:c.1427C>T:NP_009125:p.T476M\|CHEK2:NM_145862:c.1340C>T:NP_665861:p.T447M |
| F712260238 | chr7 | 6043666 | 6043666 | C | T | chr7:g.6043666C>T (NC_000007.13) | 53/591 (8.97%) | 29/1076 (2.70%) | 0.0001 |  | rs772216832 | nonsynonymous SNV | PMS2:NM_000535:c.187G>A:NP_000526:p.V63M\|PMS2:NM_001322006:c.187G>A:NP_001308935:p.V63M\|PMS2:NM_001322014:c.187G>A:NP_001308943:p.V63M |
| F712260239 | chr17 | 29528142 | 29528142 | T | C | chr17:g.29528142T>C (NC_000017.10) | 26/621 (4.19%) | 24/994 (2.41%) |  |  |  | nonsynonymous SNV | NF1:NM_000267:c.1150T>C:NP_000258:p.F384L\|NF1:NM_001042492:c.1150T>C:NP_001035957:p.F384L\|NF1:NM_001128147:c.1150T>C:NP_001121619:p.F384L |
| F712260239 | chr17 | 29541476 | 29541476 | C | T | chr17:g.29541476C>T (NC_000017.10) | 40/524 (7.63%) | 14/646 (2.17%) |  |  |  | nonsynonymous SNV | NF1:NM_000267:c.1400C>T:NP_000258:p.T467I\|NF1:NM_001042492:c.1400C>T:NP_001035957:p.T467I\|NF1:NM_001128147:c.1400C>T:NP_001121619:p.T467I |
| F712260239 | chr20 | 31022581 | 31022581 | C | A | chr20:g.31022581C>A (NC_000020.10) | 39/1265 (3.08%) | 10/779 (1.28%) |  |  |  | stopgain | ASXL1:NM_015338:c.2066C>A:NP_056153:p.S689X |
| F712260239 | chr22 | 29090054 | 29090054 | G | A | chr22:g.29090054G>A (NC_000022.10) | 102/1369 (7.45%) | 13/858 (1.52%) | 0.001 | Conflicting_interpretations_of_pathogenicity | rs142763740 | nonsynonymous SNV | CHEK2:NM_001005735:c.1556C>T:NP_001005735:p.T519M\|CHEK2:NM_001257387:c.764C>T:NP_001244316:p.T255M\|CHEK2:NM_007194:c.1427C>T:NP_009125:p.T476M\|CHEK2:NM_145862:c.1340C>T:NP_665861:p.T447M |
| F712260239 | chr7 | 6043666 | 6043666 | C | T | chr7:g.6043666C>T (NC_000007.13) | 55/775 (7.10%) | 18/953 (1.89%) | 0.0001 |  | rs772216832 | nonsynonymous SNV | PMS2:NM_000535:c.187G>A:NP_000526:p.V63M\|PMS2:NM_001322006:c.187G>A:NP_001308935:p.V63M\|PMS2:NM_001322014:c.187G>A:NP_001308943:p.V63M |
| F712260240 | chr17 | 29527569 | 29527569 | T | G | chr17:g.29527569T>G (NC_000017.10) | 14/721 (1.94%) | 12/767 (1.56%) |  |  |  | nonsynonymous SNV | NF1:NM_000267:c.1018T>G:NP_000258:p.S340A\|NF1:NM_001042492:c.1018T>G:NP_001035957:p.S340A\|NF1:NM_001128147:c.1018T>G:NP_001121619:p.S340A |
| F712260240 | chr17 | 29528142 | 29528142 | T | C | chr17:g.29528142T>C (NC_000017.10) | 23/585 (3.93%) | 13/649 (2.00%) |  |  |  | nonsynonymous SNV | NF1:NM_000267:c.1150T>C:NP_000258:p.F384L\|NF1:NM_001042492:c.1150T>C:NP_001035957:p.F384L\|NF1:NM_001128147:c.1150T>C:NP_001121619:p.F384L |
| F712260240 | chr2 | 25457242 | 25457242 | C | T | chr2:g.25457242C>T (NC_000002.11) | 69/928 (7.44%) | 25/656 (3.81%) | 0.0009 | Conflicting_interpretations_of_pathogenicity | rs147001633 | nonsynonymous SNV | DNMT3A:NM_001320893:c.2189G>A:NP_001307822:p.R730H\|DNMT3A:NM_022552:c.2645G>A:NP_072046:p.R882H\|DNMT3A:NM_153759:c.2078G>A:NP_715640:p.R693H\|DNMT3A:NM_175629:c.2645G>A:NP_783328:p.R882H |
| F712260240 | chr22 | 23651650 | 23651650 | A | G | chr22:g.23651650A>G (NC_000022.10) | 13/578 (2.25%) | 8/560 (1.43%) | 0.0002 |  | rs746213513 | nonsynonymous SNV | BCR:NM_004327:c.3052A>G:NP_004318:p.T1018A\|BCR:NM_021574:c.2920A>G:NP_067585:p.T974A |
| F712260240 | chr22 | 29090054 | 29090054 | G | A | chr22:g.29090054G>A (NC_000022.10) | 58/893 (6.49%) | 15/551 (2.72%) | 0.001 | Conflicting_interpretations_of_pathogenicity | rs142763740 | nonsynonymous SNV | CHEK2:NM_001005735:c.1556C>T:NP_001005735:p.T519M\|CHEK2:NM_001257387:c.764C>T:NP_001244316:p.T255M\|CHEK2:NM_007194:c.1427C>T:NP_009125:p.T476M\|CHEK2:NM_145862:c.1340C>T:NP_665861:p.T447M |
| F712260240 | chr5 | 131931451 | 131931451 | - | A | chr5:g.131931451T>+A (NC_000005.9) | 8/575 (1.39%) | 10/537 (1.86%) | 0.0034 | Pathogenic | rs397507178 | frameshift insertion | RAD50:NM_005732:c.2157dupA:NP_005723:p.L719fs |
| F712260240 | chr7 | 6043666 | 6043666 | C | T | chr7:g.6043666C>T (NC_000007.13) | 27/761 (3.55%) | 19/696 (2.73%) | 0.0001 |  | rs772216832 | nonsynonymous SNV | PMS2:NM_000535:c.187G>A:NP_000526:p.V63M\|PMS2:NM_001322006:c.187G>A:NP_001308935:p.V63M\|PMS2:NM_001322014:c.187G>A:NP_001308943:p.V63M |
| F712260241 | chr17 | 29527569 | 29527569 | T | G | chr17:g.29527569T>G (NC_000017.10) | 16/565 (2.83%) | 16/1230 (1.30%) |  |  |  | nonsynonymous SNV | NF1:NM_000267:c.1018T>G:NP_000258:p.S340A\|NF1:NM_001042492:c.1018T>G:NP_001035957:p.S340A\|NF1:NM_001128147:c.1018T>G:NP_001121619:p.S340A |
| F712260241 | chr17 | 29528142 | 29528142 | T | C | chr17:g.29528142T>C (NC_000017.10) | 9/548 (1.64%) | 31/1113 (2.79%) |  |  |  | nonsynonymous SNV | NF1:NM_000267:c.1150T>C:NP_000258:p.F384L\|NF1:NM_001042492:c.1150T>C:NP_001035957:p.F384L\|NF1:NM_001128147:c.1150T>C:NP_001121619:p.F384L |
| F712260241 | chr17 | 30264485 | 30264485 | C | T | chr17:g.30264485C>T (NC_000017.10) | 17/662 (2.57%) | 7/633 (1.11%) |  |  |  | nonsynonymous SNV | SUZ12:NM_001321207:c.220C>T:NP_001308136:p.P74S\|SUZ12:NM_015355:c.220C>T:NP_056170:p.P74S |
| F712260241 | chr22 | 29090054 | 29090054 | G | A | chr22:g.29090054G>A (NC_000022.10) | 72/1056 (6.82%) | 22/962 (2.29%) | 0.001 | Conflicting_interpretations_of_pathogenicity | rs142763740 | nonsynonymous SNV | CHEK2:NM_001005735:c.1556C>T:NP_001005735:p.T519M\|CHEK2:NM_001257387:c.764C>T:NP_001244316:p.T255M\|CHEK2:NM_007194:c.1427C>T:NP_009125:p.T476M\|CHEK2:NM_145862:c.1340C>T:NP_665861:p.T447M |
| F712260241 | chr3 | 10107113 | 10107113 | G | A | chr3:g.10107113G>A (NC_000003.11) | 36/510 (7.06%) | 7/587 (1.19%) | 0.0002 |  | rs755975980 | nonsynonymous SNV | FANCD2:NM_001018115:c.2204G>A:NP_001018125:p.R735Q\|FANCD2:NM_001319984:c.2204G>A:NP_001306913:p.R735Q\|FANCD2:NM_033084:c.2204G>A:NP_149075:p.R735Q |
| F712260241 | chr7 | 6043666 | 6043666 | C | T | chr7:g.6043666C>T (NC_000007.13) | 33/781 (4.23%) | 28/1109 (2.52%) | 0.0001 |  | rs772216832 | nonsynonymous SNV | PMS2:NM_000535:c.187G>A:NP_000526:p.V63M\|PMS2:NM_001322006:c.187G>A:NP_001308935:p.V63M\|PMS2:NM_001322014:c.187G>A:NP_001308943:p.V63M |
| F712280242 | chr12 | 49445038 | 49445038 | T | A | chr12:g.49445038T>A (NC_000012.11) | 17/809 (2.10%) | 12/876 (1.37%) |  |  |  | nonsynonymous SNV | KMT2D:NM_003482:c.2428A>T:NP_003473:p.T810S |
| F712280242 | chr17 | 29527569 | 29527569 | T | G | chr17:g.29527569T>G (NC_000017.10) | 19/836 (2.27%) | 18/1576 (1.14%) |  |  |  | nonsynonymous SNV | NF1:NM_000267:c.1018T>G:NP_000258:p.S340A\|NF1:NM_001042492:c.1018T>G:NP_001035957:p.S340A\|NF1:NM_001128147:c.1018T>G:NP_001121619:p.S340A |
| F712280242 | chr17 | 29528142 | 29528142 | T | C | chr17:g.29528142T>C (NC_000017.10) | 22/506 (4.35%) | 16/1164 (1.37%) |  |  |  | nonsynonymous SNV | NF1:NM_000267:c.1150T>C:NP_000258:p.F384L\|NF1:NM_001042492:c.1150T>C:NP_001035957:p.F384L\|NF1:NM_001128147:c.1150T>C:NP_001121619:p.F384L |
| F712280242 | chr17 | 29541476 | 29541476 | C | T | chr17:g.29541476C>T (NC_000017.10) | 30/525 (5.71%) | 38/1151 (3.30%) |  |  |  | nonsynonymous SNV | NF1:NM_000267:c.1400C>T:NP_000258:p.T467I\|NF1:NM_001042492:c.1400C>T:NP_001035957:p.T467I\|NF1:NM_001128147:c.1400C>T:NP_001121619:p.T467I |
| F712280242 | chr22 | 29090054 | 29090054 | G | A | chr22:g.29090054G>A (NC_000022.10) | 37/829 (4.46%) | 71/1384 (5.13%) | 0.001 | Conflicting_interpretations_of_pathogenicity | rs142763740 | nonsynonymous SNV | CHEK2:NM_001005735:c.1556C>T:NP_001005735:p.T519M\|CHEK2:NM_001257387:c.764C>T:NP_001244316:p.T255M\|CHEK2:NM_007194:c.1427C>T:NP_009125:p.T476M\|CHEK2:NM_145862:c.1340C>T:NP_665861:p.T447M |
| F712280242 | chr7 | 6043666 | 6043666 | C | T | chr7:g.6043666C>T (NC_000007.13) | 75/798 (9.40%) | 24/1457 (1.65%) | 0.0001 |  | rs772216832 | nonsynonymous SNV | PMS2:NM_000535:c.187G>A:NP_000526:p.V63M\|PMS2:NM_001322006:c.187G>A:NP_001308935:p.V63M\|PMS2:NM_001322014:c.187G>A:NP_001308943:p.V63M |
| F712280243 | chr12 | 49445038 | 49445038 | T | A | chr12:g.49445038T>A (NC_000012.11) | 8/521 (1.54%) | 11/895 (1.23%) |  |  |  | nonsynonymous SNV | KMT2D:NM_003482:c.2428A>T:NP_003473:p.T810S |
| F712280243 | chr22 | 29090054 | 29090054 | G | A | chr22:g.29090054G>A (NC_000022.10) | 33/647 (5.10%) | 80/1384 (5.78%) | 0.001 | Conflicting_interpretations_of_pathogenicity | rs142763740 | nonsynonymous SNV | CHEK2:NM_001005735:c.1556C>T:NP_001005735:p.T519M\|CHEK2:NM_001257387:c.764C>T:NP_001244316:p.T255M\|CHEK2:NM_007194:c.1427C>T:NP_009125:p.T476M\|CHEK2:NM_145862:c.1340C>T:NP_665861:p.T447M |
| F712280243 | chr7 | 6043666 | 6043666 | C | T | chr7:g.6043666C>T (NC_000007.13) | 48/590 (8.14%) | 23/1398 (1.65%) | 0.0001 |  | rs772216832 | nonsynonymous SNV | PMS2:NM_000535:c.187G>A:NP_000526:p.V63M\|PMS2:NM_001322006:c.187G>A:NP_001308935:p.V63M\|PMS2:NM_001322014:c.187G>A:NP_001308943:p.V63M |
| F801030003 | chr12 | 49435186 | 49435186 | T | G | chr12:g.49435186T>G (NC_000012.11) | 14/758 (1.85%) | 8/780 (1.03%) |  |  |  | nonsynonymous SNV | KMT2D:NM_003482:c.6367A>C:NP_003473:p.T2123P |
| F801030003 | chr12 | 49445563 | 49445563 | T | G | chr12:g.49445563T>G (NC_000012.11) | 32/645 (4.96%) | 19/414 (4.59%) |  |  |  | nonsynonymous SNV | KMT2D:NM_003482:c.1903A>C:NP_003473:p.M635L |
| F801030003 | chr17 | 29527569 | 29527569 | T | G | chr17:g.29527569T>G (NC_000017.10) | 25/1584 (1.58%) | 27/1950 (1.38%) |  |  |  | nonsynonymous SNV | NF1:NM_000267:c.1018T>G:NP_000258:p.S340A\|NF1:NM_001042492:c.1018T>G:NP_001035957:p.S340A\|NF1:NM_001128147:c.1018T>G:NP_001121619:p.S340A |
| F801030003 | chr17 | 29528142 | 29528142 | T | C | chr17:g.29528142T>C (NC_000017.10) | 14/1028 (1.36%) | 22/1533 (1.44%) |  |  |  | nonsynonymous SNV | NF1:NM_000267:c.1150T>C:NP_000258:p.F384L\|NF1:NM_001042492:c.1150T>C:NP_001035957:p.F384L\|NF1:NM_001128147:c.1150T>C:NP_001121619:p.F384L |
| F801030003 | chr17 | 29541476 | 29541476 | C | T | chr17:g.29541476C>T (NC_000017.10) | 29/920 (3.15%) | 54/1388 (3.89%) |  |  |  | nonsynonymous SNV | NF1:NM_000267:c.1400C>T:NP_000258:p.T467I\|NF1:NM_001042492:c.1400C>T:NP_001035957:p.T467I\|NF1:NM_001128147:c.1400C>T:NP_001121619:p.T467I |
| F801030003 | chr17 | 29554589 | 29554589 | C | T | chr17:g.29554589C>T (NC_000017.10) | 99/539 (18.37%) | 148/958 (15.45%) |  | Uncertain_significance | | nonsynonymous SNV | NF1:NM_000267:c.2374C>T:NP_000258:p.L792F\|NF1:NM_001042492:c.2374C>T:NP_001035957:p.L792F |
| F801030003 | chr17 | 30303572 | 30303572 | C | T | chr17:g.30303572C>T (NC_000017.10) | 22/744 (2.96%) | 27/1347 (2.00%) |  |  | rs372162318 | stopgain | SUZ12:NM_001321207:c.787C>T:NP_001308136:p.R263X\|SUZ12:NM_015355:c.856C>T:NP_056170:p.R286X |
| F801030003 | chr17 | 70119704 | 70119704 | A | C | chr17:g.70119704A>C (NC_000017.10) | 12/647 (1.85%) | 7/364 (1.92%) |  |  |  | nonsynonymous SNV | SOX9:NM_000346:c.706A>C:NP_000337:p.T236P |
| F801030003 | chr17 | 70120038 | 70120038 | A | C | chr17:g.70120038A>C (NC_000017.10) | 15/620 (2.42%) | 13/710 (1.83%) |  |  |  | nonsynonymous SNV | SOX9:NM_000346:c.1040A>C:NP_000337:p.Q347P |
| F801030003 | chr17 | 70120041 | 70120041 | A | C | chr17:g.70120041A>C (NC_000017.10) | 11/561 (1.96%) | 7/665 (1.05%) |  |  |  | nonsynonymous SNV | SOX9:NM_000346:c.1043A>C:NP_000337:p.Q348P |
| F801030003 | chr2 | 212578369 | 212578369 | G | T | chr2:g.212578369G>T (NC_000002.11) | 26/931 (2.79%) | 29/948 (3.06%) |  |  |  | nonsynonymous SNV | ERBB4:NM_001042599:c.888C>A:NP_001036064:p.N296K\|ERBB4:NM_005235:c.888C>A:NP_005226:p.N296K |
| F801030003 | chr21 | 44513243 | 44513243 | G | A | chr21:g.44513243G>A (NC_000021.8) | 9/831 (1.08%) | 14/924 (1.52%) | 0.0006 |  | rs200044775 | nonsynonymous SNV | U2AF1:NM_001025203:c.692C>T:NP_001020374:p.S231L\|U2AF1:NM_001025204:c.473C>T:NP_001020375:p.S158L\|U2AF1:NM_006758:c.692C>T:NP_006749:p.S231L\|U2AF1L5:NM_001320646:c.692C>T:NP_001307575:p.S231L\|U2AF1L5:NM_001320648:c.692C>T:NP_001307577:p.S231L\|U2AF1L5:NM_001320650:c.578C>T:NP_001307579:p.S193L\|U2AF1L5:NM_001320651:c.473C>T:NP_001307580:p.S158L |
| F801030003 | chr22 | 29090054 | 29090054 | G | A | chr22:g.29090054G>A (NC_000022.10) | 93/2007 (4.63%) | 102/1829 (5.58%) | 0.001 | Conflicting_interpretations_of_pathogenicity | rs142763740 | nonsynonymous SNV | CHEK2:NM_001005735:c.1556C>T:NP_001005735:p.T519M\|CHEK2:NM_001257387:c.764C>T:NP_001244316:p.T255M\|CHEK2:NM_007194:c.1427C>T:NP_009125:p.T476M\|CHEK2:NM_145862:c.1340C>T:NP_665861:p.T447M |
| F801030003 | chr3 | 10107113 | 10107113 | G | A | chr3:g.10107113G>A (NC_000003.11) | 25/966 (2.59%) | 27/1173 (2.30%) | 0.0002 |  | rs755975980 | nonsynonymous SNV | FANCD2:NM_001018115:c.2204G>A:NP_001018125:p.R735Q\|FANCD2:NM_001319984:c.2204G>A:NP_001306913:p.R735Q\|FANCD2:NM_033084:c.2204G>A:NP_149075:p.R735Q |
| F801030003 | chr7 | 6043613 | 6043613 | G | A | chr7:g.6043613G>A (NC_000007.13) | 57/741 (7.69%) | 33/1341 (2.46%) | 0.0011 | Conflicting_interpretations_of_pathogenicity | rs143162541 | stopgain | PMS2:NM_001322007:c.25C>T:NP_001308936:p.R9X\|PMS2:NM_001322008:c.25C>T:NP_001308937:p.R9X |
| F801030003 | chr7 | 6043666 | 6043666 | C | T | chr7:g.6043666C>T (NC_000007.13) | 65/1287 (5.05%) | 57/1891 (3.01%) | 0.0001 |  | rs772216832 | nonsynonymous SNV | PMS2:NM_000535:c.187G>A:NP_000526:p.V63M\|PMS2:NM_001322006:c.187G>A:NP_001308935:p.V63M\|PMS2:NM_001322014:c.187G>A:NP_001308943:p.V63M |
| F801030003 | chr7 | 151945291 | 151945291 | G | A | chr7:g.151945291G>A (NC_000007.13) | 60/1387 (4.33%) | 66/1977 (3.34%) | 0.0005 |  | rs763642306 | nonsynonymous SNV | KMT2C:NM_170606:c.2228C>T:NP_733751:p.P743L |
| F801250010 | chr17 | 58740809 | 58740809 | C | T | chr17:g.58740809C>T (NC_000017.10) | 97/609 (15.93%) | 318/3257 (9.76%) | 0.0011 |  | rs765769406 | stopgain | PPM1D:NM_003620:c.1714C>T:NP_003611:p.R572X |
| F801290012 | chr7 | 151970889 | 151970889 | T | C | chr7:g.151970889T>C (NC_000007.13) | 72/1876 (3.84%) | 153/4335 (3.53%) |  |  |  | nonsynonymous SNV | KMT2C:NM_170606:c.913A>G:NP_733751:p.M305V |
| F803130025 | chr5 | 131931451 | 131931451 | - | A | chr5:g.131931451T>+A (NC_000005.9) | 8/707 (1.13%) | 5/318 (1.57%) | 0.0034 | Pathogenic | rs397507178 | frameshift insertion | RAD50:NM_005732:c.2157dupA:NP_005723:p.L719fs |
| F809040100 | chr7 | 101891925 | 101891925 | A | C | chr7:g.101891925A>C (NC_000007.13) | 9/746 (1.19%) | 17/667 (2.49%) |  |  |  | nonsynonymous SNV | CUX1:NM_001202543:c.4154A>C:NP_001189472:p.E1385A\|CUX1:NM_181552:c.4121A>C:NP_853530:p.E1374A |
| F809040101 | chrX | 48650296 | 48650296 | G | A | chrX:g.48650296G>A (NC_000023.10) | 15/537 (2.72%) | 10/334 (2.91%) | 0.0003 |  | rs781920985 | nonsynonymous SNV | GATA1:NM_002049:c.266G>A:NP_002040:p.G89E |
| F903310096 | chr19 | 36219041 | 36219041 | A | G | chr19:g.36219041A>G (NC_000019.9) | 26/795 (3.27%) | 5/183 (2.73%) |  |  |  | nonsynonymous SNV | KMT2B:NM_014727:c.4540A>G:NP_055542:p.K1514E |
| F905090118 | chr6 | 157099981 | 157099981 | - | GGC | chr6:g.157099981G>+GGC (NC_000006.11) | 86/519 (16.57%) | 77/368 (20.92%) | 0.0019 | Benign/Likely_benign | rs797045286 | nonframeshift insertion | ARID1B:NM_017519:c.918_919insGGC:NP_059989:p.A306delinsAG\|ARID1B:NM_020732:c.918_919insGGC:NP_065783:p.A306delinsAG |
| F905090119 | chr17 | 17119697 | 17119697 | A | C | chr17:g.17119697A>C (NC_000017.10) | 9/508 (1.77%) | 5/342 (1.46%) |  |  |  | nonsynonymous SNV | FLCN:NM_144997:c.1297T>G:NP_659434:p.S433A |
| F908070187 | chr1 | 120539827 | 120539827 | C | T | chr1:g.120539827C>T (NC_000001.10) | 11/972 (1.13%) | 6/332 (1.81%) | 0.0001 |  | rs782038512 | nonsynonymous SNV | NOTCH2:NM_001200001:c.544G>A:NP_001186930:p.D182N\|NOTCH2:NM_024408:c.544G>A:NP_077719:p.D182N |
| F908120190 | chr2 | 25467492 | 25467492 | G | C | chr2:g.25467492G>C (NC_000002.11) | 11/809 (1.36%) | 8/597 (1.34%) |  |  |  | stopgain | DNMT3A:NM_001320893:c.1128C>G:NP_001307822:p.Y376X\|DNMT3A:NM_022552:c.1584C>G:NP_072046:p.Y528X\|DNMT3A:NM_153759:c.1017C>G:NP_715640:p.Y339X\|DNMT3A:NM_175629:c.1584C>G:NP_783328:p.Y528X |
| F908180197 | chr17 | 58740836 | 58740836 | C | T | chr17:g.58740836C>T (NC_000017.10) | 19/803 (2.37%) | 3/227 (1.32%) |  |  | rs747668756 | stopgain | PPM1D:NM_003620:c.1741C>T:NP_003611:p.R581X |
| F001190024 | chr16 | 89865631 | 89865631 | T | G | chr16:g.89865631T>G (NC_000016.9) | 6/549 (1.09%) | 3/277 (1.08%) |  |  |  | nonsynonymous SNV | FANCA:NM_000135:c.836A>C:NP_000126:p.D279A\|FANCA:NM_001018112:c.836A>C:NP_001018122:p.D279A\|FANCA:NM_001286167:c.836A>C:NP_001273096:p.D279A |
