# Supplementary material for: Clinical application of liquid biopsy in cancer patients
Source: BMC Cancer. 2022 Apr 15;22:413. doi: 10.1186/s12885-022-09525-0 (PMC9011972; doi:10.1186/s12885-022-09525-0)
Supplement: Supplementary file 5 — Additional file 5: Table S5. cfDNA P/LP somatic mutations list in cancer patients. [file 12885_2022_9525_MOESM5_ESM.docx]

| Sample_ID | Cancer types | Chrom | start_pos | end_pos | ref | alt | Variants | Depth_and_Ratio in plasma | Allele_Freq | clinvar | avsnp | Type | HGVS |
| --- | --- | --- | --- | --- | --- | --- | --- | --- | --- | --- | --- | --- | --- |
| F711150221 | Ovarian | chr3 | 178952085 | 178952085 | A | G | chr3:g.178952085A>G (NC_000003.11) | 27/728 (3.71%) | 0 | Pathogenic | rs121913279 | nonsynonymous SNV | PIK3CA:NM_006218:c.3140A>G:NP_006209:p.H1047R |
| F801020001 | Colorectal | chr17 | 29664535 | 29664536 | GA | - | chr17:g.29664534T>-GA (NC_000017.10) | 6/551 (1.09%) |  | Pathogenic |  | frameshift deletion | NF1:NM_000267:c.6514_6515del:NP_000258:p.E2172fs\|NF1:NM_001042492:c.6577_6578del:NP_001035957:p.E2193fs |
| F804040035 | Ovarian | chr12 | 49426733 | 49426733 | G | A | chr12:g.49426733G>A (NC_000012.11) | 334/1176 (28.40%) |  | Pathogenic |  | stopgain | KMT2D:NM_003482:c.11755C>T:NP_003473:p.Q3919X |
| F804040035 | Ovarian | chr13 | 32953633 | 32953633 | A | - | chr13:g.32953632C>-A (NC_000013.10) | 10/903 (1.11%) |  | Pathogenic | rs1064793320 | frameshift deletion | BRCA2:NM_000059:c.8934delA:NP_000050:p.S2978fs |
| F804040035 | Ovarian | chr17 | 7578406 | 7578406 | C | T | chr17:g.7578406C>T (NC_000017.10) | 194/514 (37.74%) | 0 | Pathogenic | rs28934578 | nonsynonymous SNV | TP53:NM_000546:c.524G>A:NP_000537:p.R175H\|TP53:NM_001126112:c.524G>A:NP_001119584:p.R175H\|TP53:NM_001126113:c.524G>A:NP_001119585:p.R175H\|TP53:NM_001126114:c.524G>A:NP_001119586:p.R175H\|TP53:NM_001126115:c.128G>A:NP_001119587:p.R43H\|TP53:NM_001126116:c.128G>A:NP_001119588:p.R43H\|TP53:NM_001126117:c.128G>A:NP_001119589:p.R43H\|TP53:NM_001126118:c.407G>A:NP_001119590:p.R136H\|TP53:NM_001276695:c.407G>A:NP_001263624:p.R136H\|TP53:NM_001276696:c.407G>A:NP_001263625:p.R136H\|TP53:NM_001276697:c.47G>A:NP_001263626:p.R16H\|TP53:NM_001276698:c.47G>A:NP_001263627:p.R16H\|TP53:NM_001276699:c.47G>A:NP_001263628:p.R16H\|TP53:NM_001276760:c.407G>A:NP_001263689:p.R136H\|TP53:NM_001276761:c.407G>A:NP_001263690:p.R136H |
| F804040035 | Ovarian | chr2 | 48030639 | 48030639 | - | C | chr2:g.48030639A>+C (NC_000002.11) | 12/952 (1.26%) | 0.0052 | Pathogenic | rs267608087 | frameshift insertion | MSH6:NM_000179:c.3254dupC:NP_000170:p.T1085fs\|MSH6:NM_001281492:c.2864dupC:NP_001268421:p.T955fs\|MSH6:NM_001281493:c.2348dupC:NP_001268422:p.T783fs\|MSH6:NM_001281494:c.2348dupC:NP_001268423:p.T783fs |
| F809200112 | Colorectal | chr2 | 209113113 | 209113113 | G | A | chr2:g.209113113G>A (NC_000002.11) | 35/872 (3.86%) |  | Pathogenic/Likely_pathogenic | rs121913499 | nonsynonymous SNV | IDH1:NM_001282386:c.394C>T:NP_001269315:p.R132C\|IDH1:NM_001282387:c.394C>T:NP_001269316:p.R132C\|IDH1:NM_005896:c.394C>T:NP_005887:p.R132C |
| F809200112 | Colorectal | chr7 | 140453136 | 140453136 | A | T | chr7:g.140453136A>T (NC_000007.13) | 78/1932 (3.88%) | 0.0001 | Pathogenic | rs113488022 | nonsynonymous SNV | BRAF:NM_004333:c.1799T>A:NP_004324:p.V600E |
| F910050257 | Breast | chr2 | 48030692 | 48030692 | T | - | chr2:g.48030691C>-T (NC_000002.11) | 9/639 (1.41%) |  | Pathogenic | rs267608093 | frameshift deletion | MSH6:NM_000179:c.3306delT:NP_000170:p.T1102fs\|MSH6:NM_001281492:c.2916delT:NP_001268421:p.T972fs\|MSH6:NM_001281493:c.2400delT:NP_001268422:p.T800fs\|MSH6:NM_001281494:c.2400delT:NP_001268423:p.T800fs |
| F910310285 | Lung | chr17 | 7577580 | 7577580 | T | C | chr17:g.7577580T>C (NC_000017.10) | 267/2066 (12.92%) | 0 | Pathogenic/Likely_pathogenic | rs587780073 | nonsynonymous SNV | TP53:NM_000546:c.701A>G:NP_000537:p.Y234C\|TP53:NM_001126112:c.701A>G:NP_001119584:p.Y234C\|TP53:NM_001126113:c.701A>G:NP_001119585:p.Y234C\|TP53:NM_001126114:c.701A>G:NP_001119586:p.Y234C\|TP53:NM_001126115:c.305A>G:NP_001119587:p.Y102C\|TP53:NM_001126116:c.305A>G:NP_001119588:p.Y102C\|TP53:NM_001126117:c.305A>G:NP_001119589:p.Y102C\|TP53:NM_001126118:c.584A>G:NP_001119590:p.Y195C\|TP53:NM_001276695:c.584A>G:NP_001263624:p.Y195C\|TP53:NM_001276696:c.584A>G:NP_001263625:p.Y195C\|TP53:NM_001276697:c.224A>G:NP_001263626:p.Y75C\|TP53:NM_001276698:c.224A>G:NP_001263627:p.Y75C\|TP53:NM_001276699:c.224A>G:NP_001263628:p.Y75C\|TP53:NM_001276760:c.584A>G:NP_001263689:p.Y195C\|TP53:NM_001276761:c.584A>G:NP_001263690:p.Y195C |
| F910310285 | Lung | chr3 | 41266113 | 41266113 | C | G | chr3:g.41266113C>G (NC_000003.11) | 38/665 (5.71%) |  | Pathogenic/Likely_pathogenic | rs121913403 | nonsynonymous SNV | CTNNB1:NM_001098209:c.110C>G:NP_001091679:p.S37C\|CTNNB1:NM_001098210:c.110C>G:NP_001091680:p.S37C\|CTNNB1:NM_001330729:c.89C>G:NP_001317658:p.S30C\|CTNNB1:NM_001904:c.110C>G:NP_001895:p.S37C |
| F910310285 | Lung | chr3 | 181430286 | 181430286 | T | G | chr3:g.181430286T>G (NC_000003.11) | 8/670 (1.19%) |  | Pathogenic | rs104893806 | nonsynonymous SNV | SOX2:NM_003106:c.138T>G:NP_003097:p.N46K |
| F912080321 | Lung | chr17 | 29559144 | 29559144 | C | T | chr17:g.29559144C>T (NC_000017.10) | 10/849 (1.18%) |  | Likely_pathogenic | | nonsynonymous SNV | NF1:NM_000267:c.3251C>T:NP_000258:p.P1084L\|NF1:NM_001042492:c.3251C>T:NP_001035957:p.P1084L |
| F912080321 | Lung | chr17 | 37880984 | 37880984 | - | TACGTGATGGCT | chr17:g.37880984A>+TACGTGATGGCT (NC_000017.10) | 9/516 (1.74%) |  | Likely_pathogenic | rs397516977 | nonframeshift insertion | ERBB2:NM_001005862:c.2223_2224insTACGTGATGGCT:NP_001005862:p.A741delinsAYVMA\|ERBB2:NM_001289936:c.2268_2269insTACGTGATGGCT:NP_001276865:p.A756delinsAYVMA\|ERBB2:NM_001289937:c.2313_2314insTACGTGATGGCT:NP_001276866:p.A771delinsAYVMA\|ERBB2:NM_004448:c.2313_2314insTACGTGATGGCT:NP_004439:p.A771delinsAYVMA |
| F912150333 | Ovarian | chr17 | 7577548 | 7577548 | C | T | chr17:g.7577548C>T (NC_000017.10) | 12/542 (2.21%) | 0.0001 | Pathogenic | rs28934575 | nonsynonymous SNV | TP53:NM_000546:c.733G>A:NP_000537:p.G245S\|TP53:NM_001126112:c.733G>A:NP_001119584:p.G245S\|TP53:NM_001126113:c.733G>A:NP_001119585:p.G245S\|TP53:NM_001126114:c.733G>A:NP_001119586:p.G245S\|TP53:NM_001126115:c.337G>A:NP_001119587:p.G113S\|TP53:NM_001126116:c.337G>A:NP_001119588:p.G113S\|TP53:NM_001126117:c.337G>A:NP_001119589:p.G113S\|TP53:NM_001126118:c.616G>A:NP_001119590:p.G206S\|TP53:NM_001276695:c.616G>A:NP_001263624:p.G206S\|TP53:NM_001276696:c.616G>A:NP_001263625:p.G206S\|TP53:NM_001276697:c.256G>A:NP_001263626:p.G86S\|TP53:NM_001276698:c.256G>A:NP_001263627:p.G86S\|TP53:NM_001276699:c.256G>A:NP_001263628:p.G86S\|TP53:NM_001276760:c.616G>A:NP_001263689:p.G206S\|TP53:NM_001276761:c.616G>A:NP_001263690:p.G206S |
| F912290349 | Lung | chr7 | 55259524 | 55259524 | T | A | chr7:g.55259524T>A (NC_000007.13) | 210/1213 (17.31%) |  | Pathogenic/Likely_pathogenic\x2c_drug_response | rs121913444 | nonsynonymous SNV | EGFR:NM_005228:c.2582T>A:NP_005219:p.L861Q |
| F912310356 | Endometrial | chr10 | 89717650 | 89717650 | T | G | chr10:g.89717650T>G (NC_000010.10) | 1473/2507 (58.76%) |  | Pathogenic | rs1057520900 | stopgain | PTEN:NM_000314:c.675T>G:NP_000305:p.Y225X\|PTEN:NM_001304717:c.1194T>G:NP_001291646:p.Y398X\|PTEN:NM_001304718:c.84T>G:NP_001291647:p.Y28X |
| F912310356 | Endometrial | chr10 | 89720649 | 89720649 | A | T | chr10:g.89720649A>T (NC_000010.10) | 8/759 (1.05%) |  | Pathogenic | rs587782455 | splicing | PTEN(NM_001304718:exon8:c.211-2A>T,NM_001304717:exon9:c.1321-2A>T,NM_000314:exon8:c.802-2A>T) |
| F912310356 | Endometrial | chr10 | 123279677 | 123279677 | G | C | chr10:g.123279677G>C (NC_000010.10) | 763/2531 (30.15%) | 0 | Pathogenic | rs79184941 | nonsynonymous SNV | FGFR2:NM_000141:c.755C>G:NP_000132:p.S252W\|FGFR2:NM_001144913:c.755C>G:NP_001138385:p.S252W\|FGFR2:NM_001144915:c.488C>G:NP_001138387:p.S163W\|FGFR2:NM_001144916:c.410C>G:NP_001138388:p.S137W\|FGFR2:NM_001144917:c.755C>G:NP_001138389:p.S252W\|FGFR2:NM_001144918:c.410C>G:NP_001138390:p.S137W\|FGFR2:NM_001144919:c.488C>G:NP_001138391:p.S163W\|FGFR2:NM_001320654:c.71C>G:NP_001307583:p.S24W\|FGFR2:NM_001320658:c.755C>G:NP_001307587:p.S252W\|FGFR2:NM_022970:c.755C>G:NP_075259:p.S252W\|FGFR2:NM_023029:c.488C>G:NP_075418:p.S163W |
| F912310356 | Endometrial | chr12 | 49420288 | 49420288 | C | T | chr12:g.49420288C>T (NC_000012.11) | 614/1860 (33.01%) |  | Pathogenic/Likely_pathogenic | rs886043497 | nonsynonymous SNV | KMT2D:NM_003482:c.15461G>A:NP_003473:p.R5154Q |
| F912310356 | Endometrial | chr3 | 41266101 | 41266101 | C | G | chr3:g.41266101C>G (NC_000003.11) | 612/2001 (30.58%) |  | Likely_pathogenic\x2c_other | rs121913400 | nonsynonymous SNV | CTNNB1:NM_001098209:c.98C>G:NP_001091679:p.S33C\|CTNNB1:NM_001098210:c.98C>G:NP_001091680:p.S33C\|CTNNB1:NM_001330729:c.77C>G:NP_001317658:p.S26C\|CTNNB1:NM_001904:c.98C>G:NP_001895:p.S33C |
| F912310356 | Endometrial | chr3 | 178916876 | 178916876 | G | A | chr3:g.178916876G>A (NC_000003.11) | 973/4436 (21.93%) |  | Likely_pathogenic | rs121913287 | nonsynonymous SNV | PIK3CA:NM_006218:c.263G>A:NP_006209:p.R88Q |
| F001110013 | Lung | chr17 | 7578275 | 7578275 | G | A | chr17:g.7578275G>A (NC_000017.10) | 69/1564 (4.41%) |  | Pathogenic | rs866380588 | stopgain | TP53:NM_000546:c.574C>T:NP_000537:p.Q192X\|TP53:NM_001126112:c.574C>T:NP_001119584:p.Q192X\|TP53:NM_001126113:c.574C>T:NP_001119585:p.Q192X\|TP53:NM_001126114:c.574C>T:NP_001119586:p.Q192X\|TP53:NM_001126115:c.178C>T:NP_001119587:p.Q60X\|TP53:NM_001126116:c.178C>T:NP_001119588:p.Q60X\|TP53:NM_001126117:c.178C>T:NP_001119589:p.Q60X\|TP53:NM_001126118:c.457C>T:NP_001119590:p.Q153X\|TP53:NM_001276695:c.457C>T:NP_001263624:p.Q153X\|TP53:NM_001276696:c.457C>T:NP_001263625:p.Q153X\|TP53:NM_001276697:c.97C>T:NP_001263626:p.Q33X\|TP53:NM_001276698:c.97C>T:NP_001263627:p.Q33X\|TP53:NM_001276699:c.97C>T:NP_001263628:p.Q33X\|TP53:NM_001276760:c.457C>T:NP_001263689:p.Q153X\|TP53:NM_001276761:c.457C>T:NP_001263690:p.Q153X |
| F003050077 | Colorectal | chr12 | 25398284 | 25398284 | C | T | chr12:g.25398284C>T (NC_000012.11) | 316/3544 (8.92%) | 0.0001 | Pathogenic | rs121913529 | nonsynonymous SNV | KRAS:NM_004985:c.35G>A:NP_004976:p.G12D\|KRAS:NM_033360:c.35G>A:NP_203524:p.G12D |
| F003050077 | Colorectal | chr17 | 7578403 | 7578403 | C | A | chr17:g.7578403C>A (NC_000017.10) | 211/3275 (6.44%) |  | Likely_pathogenic | rs786202962 | nonsynonymous SNV | TP53:NM_000546:c.527G>T:NP_000537:p.C176F\|TP53:NM_001126112:c.527G>T:NP_001119584:p.C176F\|TP53:NM_001126113:c.527G>T:NP_001119585:p.C176F\|TP53:NM_001126114:c.527G>T:NP_001119586:p.C176F\|TP53:NM_001126115:c.131G>T:NP_001119587:p.C44F\|TP53:NM_001126116:c.131G>T:NP_001119588:p.C44F\|TP53:NM_001126117:c.131G>T:NP_001119589:p.C44F\|TP53:NM_001126118:c.410G>T:NP_001119590:p.C137F\|TP53:NM_001276695:c.410G>T:NP_001263624:p.C137F\|TP53:NM_001276696:c.410G>T:NP_001263625:p.C137F\|TP53:NM_001276697:c.50G>T:NP_001263626:p.C17F\|TP53:NM_001276698:c.50G>T:NP_001263627:p.C17F\|TP53:NM_001276699:c.50G>T:NP_001263628:p.C17F\|TP53:NM_001276760:c.410G>T:NP_001263689:p.C137F\|TP53:NM_001276761:c.410G>T:NP_001263690:p.C137F |
| F003200099 | Renal cell | chr10 | 89720649 | 89720649 | A | T | chr10:g.89720649A>T (NC_000010.10) | 7/519 (1.35%) |  | Pathogenic | rs587782455 | splicing | PTEN(NM_001304718:exon8:c.211-2A>T,NM_001304717:exon9:c.1321-2A>T,NM_000314:exon8:c.802-2A>T) |
| F004060124 | Lung | chr17 | 7577534 | 7577534 | C | A | chr17:g.7577534C>A (NC_000017.10) | 38/724 (5.25%) |  | Pathogenic(2),Uncertain_significance(2) | rs28934571 | nonsynonymous SNV | TP53:NM_000546:c.747G>T:NP_000537:p.R249S\|TP53:NM_001126112:c.747G>T:NP_001119584:p.R249S\|TP53:NM_001126113:c.747G>T:NP_001119585:p.R249S\|TP53:NM_001126114:c.747G>T:NP_001119586:p.R249S\|TP53:NM_001126115:c.351G>T:NP_001119587:p.R117S\|TP53:NM_001126116:c.351G>T:NP_001119588:p.R117S\|TP53:NM_001126117:c.351G>T:NP_001119589:p.R117S\|TP53:NM_001126118:c.630G>T:NP_001119590:p.R210S\|TP53:NM_001276695:c.630G>T:NP_001263624:p.R210S\|TP53:NM_001276696:c.630G>T:NP_001263625:p.R210S\|TP53:NM_001276697:c.270G>T:NP_001263626:p.R90S\|TP53:NM_001276698:c.270G>T:NP_001263627:p.R90S\|TP53:NM_001276699:c.270G>T:NP_001263628:p.R90S\|TP53:NM_001276760:c.630G>T:NP_001263689:p.R210S\|TP53:NM_001276761:c.630G>T:NP_001263690:p.R210S |
| F004160134 | Lung | chr17 | 7577538 | 7577538 | C | A | chr17:g.7577538C>A (NC_000017.10) | 1144/1633 (70.06%) |  | Pathogenic | rs11540652 | nonsynonymous SNV | TP53:NM_000546:c.743G>T:NP_000537:p.R248L\|TP53:NM_001126112:c.743G>T:NP_001119584:p.R248L\|TP53:NM_001126113:c.743G>T:NP_001119585:p.R248L\|TP53:NM_001126114:c.743G>T:NP_001119586:p.R248L\|TP53:NM_001126115:c.347G>T:NP_001119587:p.R116L\|TP53:NM_001126116:c.347G>T:NP_001119588:p.R116L\|TP53:NM_001126117:c.347G>T:NP_001119589:p.R116L\|TP53:NM_001126118:c.626G>T:NP_001119590:p.R209L\|TP53:NM_001276695:c.626G>T:NP_001263624:p.R209L\|TP53:NM_001276696:c.626G>T:NP_001263625:p.R209L\|TP53:NM_001276697:c.266G>T:NP_001263626:p.R89L\|TP53:NM_001276698:c.266G>T:NP_001263627:p.R89L\|TP53:NM_001276699:c.266G>T:NP_001263628:p.R89L\|TP53:NM_001276760:c.626G>T:NP_001263689:p.R209L\|TP53:NM_001276761:c.626G>T:NP_001263690:p.R209L |
| F004210140 | Lung | chr12 | 49446774 | 49446777 | AGAG | - | chr12:g.49446773C>-AGAG (NC_000012.11) | 346/1702 (20.33%) |  | Pathogenic |  | frameshift deletion | KMT2D:NM_003482:c.1033_1036del:NP_003473:p.L345fs |
| F005040165 | Lung | chr17 | 7577547 | 7577547 | C | A | chr17:g.7577547C>A (NC_000017.10) | 30/1097 (2.73%) | 0.0001 | Pathogenic/Likely_pathogenic | rs121912656 | nonsynonymous SNV | TP53:NM_000546:c.734G>T:NP_000537:p.G245V\|TP53:NM_001126112:c.734G>T:NP_001119584:p.G245V\|TP53:NM_001126113:c.734G>T:NP_001119585:p.G245V\|TP53:NM_001126114:c.734G>T:NP_001119586:p.G245V\|TP53:NM_001126115:c.338G>T:NP_001119587:p.G113V\|TP53:NM_001126116:c.338G>T:NP_001119588:p.G113V\|TP53:NM_001126117:c.338G>T:NP_001119589:p.G113V\|TP53:NM_001126118:c.617G>T:NP_001119590:p.G206V\|TP53:NM_001276695:c.617G>T:NP_001263624:p.G206V\|TP53:NM_001276696:c.617G>T:NP_001263625:p.G206V\|TP53:NM_001276697:c.257G>T:NP_001263626:p.G86V\|TP53:NM_001276698:c.257G>T:NP_001263627:p.G86V\|TP53:NM_001276699:c.257G>T:NP_001263628:p.G86V\|TP53:NM_001276760:c.617G>T:NP_001263689:p.G206V\|TP53:NM_001276761:c.617G>T:NP_001263690:p.G206V |
| F005040165 | Lung | chr3 | 178936082 | 178936082 | G | A | chr3:g.178936082G>A (NC_000003.11) | 13/1095 (1.19%) |  | Pathogenic/Likely_pathogenic | rs121913273 | nonsynonymous SNV | PIK3CA:NM_006218:c.1624G>A:NP_006209:p.E542K |
| F005040165 | Lung | chr3 | 178936091 | 178936091 | G | A | chr3:g.178936091G>A (NC_000003.11) | 13/1109 (1.17%) | 0 | Pathogenic/Likely_pathogenic | rs104886003 | nonsynonymous SNV | PIK3CA:NM_006218:c.1633G>A:NP_006209:p.E545K |
| F005040165 | Lung | chr5 | 112175021 | 112175021 | C | T | chr5:g.112175021C>T (NC_000005.9) | 20/1157 (1.73%) |  | Pathogenic | rs79122263 | stopgain | APC:NM_000038:c.3730C>T:NP_000029:p.Q1244X\|APC:NM_001127510:c.3730C>T:NP_001120982:p.Q1244X\|APC:NM_001127511:c.3676C>T:NP_001120983:p.Q1226X |
| F007010246 | Lung | chr17 | 29559144 | 29559144 | C | T | chr17:g.29559144C>T (NC_000017.10) | 7/632 (1.11%) |  | Likely_pathogenic | rs1164081667 | nonsynonymous SNV | NF1:NM_000267:c.3251C>T:NP_000258:p.P1084L\|NF1:NM_001042492:c.3251C>T:NP_001035957:p.P1084L |
| F006180223 | Endometrial | chr3 | 181430286 | 181430286 | T | G | chr3:g.181430286T>G (NC_000003.11) | 6/572 (1.05%) |  | Pathogenic | rs104893806 | nonsynonymous SNV | SOX2:NM_003106:c.138T>G:NP_003097:p.N46K |
| F006090212 | Ovarian | chr10 | 123276955 | 123276955 | T | G | chr10:g.123276955T>G (NC_000010.10) | 6/554 (1.08%) |  | Pathogenic | rs121918510 | nonsynonymous SNV | FGFR2:NM_000141:c.962A>C:NP_000132:p.D321A\|FGFR2:NM_001144915:c.695A>C:NP_001138387:p.D232A\|FGFR2:NM_001144916:c.617A>C:NP_001138388:p.D206A\|FGFR2:NM_001144918:c.617A>C:NP_001138390:p.D206A\|FGFR2:NM_001320654:c.278A>C:NP_001307583:p.D93A\|FGFR2:NM_001320658:c.962A>C:NP_001307587:p.D321A\|FGFR2:NM_023029:c.695A>C:NP_075418:p.D232A |
| F808060081 | Ovarian | chr17 | 7577120 | 7577120 | C | A | chr17:g.7577120C>A (NC_000017.10) | 28/583 (4.80%) | 0 | Pathogenic | rs28934576 | nonsynonymous SNV | TP53:NM_000546:c.818G>T:NP_000537:p.R273L\|TP53:NM_001126112:c.818G>T:NP_001119584:p.R273L\|TP53:NM_001126113:c.818G>T:NP_001119585:p.R273L\|TP53:NM_001126114:c.818G>T:NP_001119586:p.R273L\|TP53:NM_001126115:c.422G>T:NP_001119587:p.R141L\|TP53:NM_001126116:c.422G>T:NP_001119588:p.R141L\|TP53:NM_001126117:c.422G>T:NP_001119589:p.R141L\|TP53:NM_001126118:c.701G>T:NP_001119590:p.R234L\|TP53:NM_001276695:c.701G>T:NP_001263624:p.R234L\|TP53:NM_001276696:c.701G>T:NP_001263625:p.R234L\|TP53:NM_001276697:c.341G>T:NP_001263626:p.R114L\|TP53:NM_001276698:c.341G>T:NP_001263627:p.R114L\|TP53:NM_001276699:c.341G>T:NP_001263628:p.R114L\|TP53:NM_001276760:c.701G>T:NP_001263689:p.R234L\|TP53:NM_001276761:c.701G>T:NP_001263690:p.R234L |
| F003200099 | Renal cell | chr1 | 156830727 | 156830727 | A | C | chr1:g.156830727A>C (NC_000001.10) | 7/591 (1.18%) |  | Pathogenic |  | nonsynonymous SNV | NTRK1:NM_001012331:c.1A>C:NP_001012331:p.M1L\|NTRK1:NM_002529:c.1A>C:NP_002520:p.M1L |
| F003200099 | Renal cell | chr3 | 181430286 | 181430286 | T | G | chr3:g.181430286T>G (NC_000003.11) | 7/546 (1.28%) |  | Pathogenic | rs104893806 | nonsynonymous SNV | SOX2:NM_003106:c.138T>G:NP_003097:p.N46K |
