# Supplementary material for: Clinical application of liquid biopsy in cancer patients
Source: BMC Cancer. 2022 Apr 15;22:413. doi: 10.1186/s12885-022-09525-0 (PMC9011972; doi:10.1186/s12885-022-09525-0)
Supplement: Supplementary file 6 — Additional file 6: Table S6. cfDNA P/LP somatic mutations list in healthy individuals. [file 12885_2022_9525_MOESM6_ESM.docx]

| Sample_ID | Chrom | start_pos | end_pos | ref | alt | Variants | Depth_and_Ratio in plasma | Allele_Freq | clinvar | avsnp | Type | HGVS |
| --- | --- | --- | --- | --- | --- | --- | --- | --- | --- | --- | --- | --- |
| F803130025 | chr8 | 128750693 | 128750693 | C | T | chr8:g.128750693C>T (NC_000008.10) | 9/880 (1.02%) |  | Likely_pathogenic | rs1057519850 | nonsynonymous SNV | MYC:NM_002467:c.230C>T:NP_002458:p.S77F |
