# Supplementary material for: Clinical application of liquid biopsy in cancer patients
Source: BMC Cancer. 2022 Apr 15;22:413. doi: 10.1186/s12885-022-09525-0 (PMC9011972; doi:10.1186/s12885-022-09525-0)
Supplement: Supplementary file 7 — Additional file 7: Table S7. cfDNA P/LP germline mutations list in cancer patients. [file 12885_2022_9525_MOESM7_ESM.docx]

| Sample_ID | Cancer types | Chrom | start_pos | end_pos | ref | alt | Variants | Depth_and_Ratio in plasma | Depth_and_Ratio in WBCs | Allele_Freq | clinvar | avsnp | Type | HGVS |
| --- | --- | --- | --- | --- | --- | --- | --- | --- | --- | --- | --- | --- | --- | --- |
| F808070084 | Ovarian | chr2 | 47703631 | 47703631 | C | T | chr2:g.47703631C>T (NC_000002.11) | 155/349 (44.41%) | 214/416 (51.44%) |  | Pathogenic | rs63750636 | stopgain | MSH2:NM_000251:c.2131C>T:NP_000242:p.R711X\|MSH2:NM_001258281:c.1933C>T:NP_001245210:p.R645X |
| F902260081 | Ovarian | chr17 | 41219627 | 41219627 | G | T | chr17:g.41219627G>T (NC_000017.10) | 1610/3318 (48.52%) | 227/537 (42.27%) |  | Likely_pathogenic | rs80357034 | nonsynonymous SNV | BRCA1:NM_007294:c.5072C>A:NP_009225:p.T1691K\|BRCA1:NM_007297:c.4931C>A:NP_009228:p.T1644K\|BRCA1:NM_007298:c.1760C>A:NP_009229:p.T587K\|BRCA1:NM_007299:c.1760C>A:NP_009230:p.T587K\|BRCA1:NM_007300:c.5135C>A:NP_009231:p.T1712K |
| F905070116 | Ovarian | chr1 | 45799108 | 45799108 | G | A | chr1:g.45799108G>A (NC_000001.10) | 229/445 (51.46%) | 132/272 (48.53%) | 0.0001 | Pathogenic/Likely_pathogenic | rs765123255 | nonsynonymous SNV | MUTYH:NM_001048171:c.283C>T:NP_001041636:p.R95W\|MUTYH:NM_001048172:c.244C>T:NP_001041637:p.R82W\|MUTYH:NM_001048173:c.241C>T:NP_001041638:p.R81W\|MUTYH:NM_001048174:c.241C>T:NP_001041639:p.R81W\|MUTYH:NM_001128425:c.325C>T:NP_001121897:p.R109W\|MUTYH:NM_001293190:c.286C>T:NP_001280119:p.R96W\|MUTYH:NM_001293191:c.274C>T:NP_001280120:p.R92W\|MUTYH:NM_001293195:c.241C>T:NP_001280124:p.R81W\|MUTYH:NM_012222:c.316C>T:NP_036354:p.R106W |
| F808030080 | Cervical | chr5 | 131931451 | 131931451 | - | A | chr5:g.131931451T>+A (NC_000005.9) | 147/378 (38.89%) | 713/1126 (38.77%) | 0.0034 | Pathogenic | rs397507178 | frameshift insertion | RAD50:NM_005732:c.2157dupA:NP_005723:p.L719fs |
| F001040002 | Endometrial | chr13 | 32907376 | 32907379 | AAAT | - | chr13:g.32907375C>-AAAT (NC_000013.10) | 264/570 (46.32%) | 97/227 (42.73%) |  | Pathogenic | rs80359303 | frameshift deletion | BRCA2:NM_000059:c.1761_1764del:NP_000050:p.T587fs |
| F912080320 | Lung | chr17 | 59871088 | 59871088 | C | T | chr17:g.59871088C>T (NC_000017.10) | 287/542 (52.95%) | 76/173 (43.93%) | 0.0005 | Pathogenic/Likely_pathogenic | rs775171520 | stopgain | BRIP1:NM_032043:c.1343G>A:NP_114432:p.W448X |
| F002080045 | Lung | chr1 | 43812115 | 43812115 | G | C | chr1:g.43812115G>C (NC_000001.10) | 360/720 (50.00%) | 543/1305 (41.61%) | 0.0001 | Likely_pathogenic | rs769297582 | splicing | MPL(NM_005373:exon7:c.981-1G>C) |
