# Supplementary material for: Clinical application of liquid biopsy in cancer patients
Source: BMC Cancer. 2022 Apr 15;22:413. doi: 10.1186/s12885-022-09525-0 (PMC9011972; doi:10.1186/s12885-022-09525-0)
Supplement: Supplementary file 8 — Additional file 8: Table S8. cfDNA P/LP germline mutations list in healthy individuals. [file 12885_2022_9525_MOESM8_ESM.docx]

| Sample_ID | Chrom | start_pos | end_pos | ref | alt | Variants | Depth_and_Ratio in plasma | Depth_and_Ratio in WBCs | Allele_Freq | clinvar | avsnp | Type | HGVS |
| --- | --- | --- | --- | --- | --- | --- | --- | --- | --- | --- | --- | --- | --- |
| F809040100 | chr19 | 15298126 | 15298126 | G | A | chr19:g.15298126G>A (NC_000019.9) | 247/274 (47.41%) | 258/268 (49.05%) | 0.0036 | Likely_pathogenic | rs201118034 | nonsynonymous SNV | NOTCH3:NM_000435:c.1630C>T:NP_000426:p.R544C |
