# Supplementary material for: Clinical application of liquid biopsy in cancer patients
Source: BMC Cancer. 2022 Apr 15;22:413. doi: 10.1186/s12885-022-09525-0 (PMC9011972; doi:10.1186/s12885-022-09525-0)
Supplement: Supplementary file 9 — Additional file 9: Table S9. Characteristics of next-generation sequencing outcomes of FFPE and cfDNA in different time. [file 12885_2022_9525_MOESM9_ESM.docx]

| Cancer types | Sample ID | FFPE | Depth_and_Ratio in FFPE | cfDNA | Depth_and_Ratio in plasma |
| --- | --- | --- | --- | --- | --- |
| colorectal cancer | F809260123 | *TP53* c.610G>T; p.E204X | 301/967 (31.13%) | na |  |
|  |  | *APC* c.646C>T; p.R216X | 166/793 (20.93%) | na |  |
| urothelial carcinoma | F901020002 | na |  | na |  |
| pancreatic cancer | F902200079 | na |  | na |  |
| ovarian cancer | F906220139 | na |  | na |  |
| ovarian cancer | F912310359 | na |  | na |  |
| head and neck | F908280208 | na |  | na |  |
| lung cancer | F004060124 | *TP53* c.747G>T; p.R249S | 12/891 (1.35%) | *TP53* c.747G>T; p.R249S | 38/724 (5.25%) |
| lung cancer | F004160134 | *TP53* c.743G>T; p.R248L | 473/780 (60.64%) | *TP53* c.743G>T; p.R248L | 1144/1633 (70.06%) |
| lung cancer | F004280146 | na |  | na |  |
