# Supplementary figures and images for: Clinical application of liquid biopsy in cancer patients
Source: BMC Cancer. 2022 Apr 15;22:413. doi: 10.1186/s12885-022-09525-0 (PMC9011972; doi:10.1186/s12885-022-09525-0)

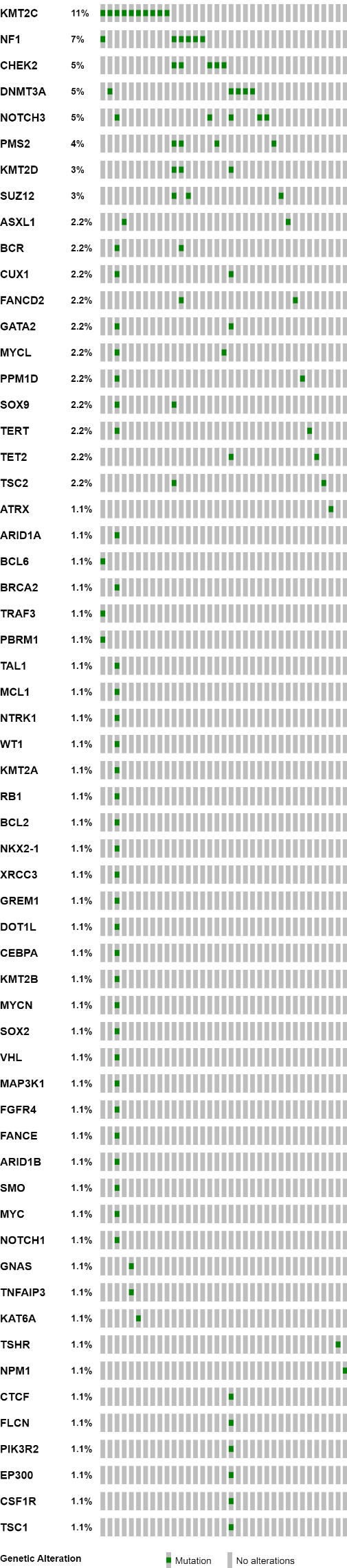

Supplement: Supplementary file 10 — Additional file 10: Figure S1. Oncoprint showing the distribution of CH genes in cancer patients. [file 12885_2022_9525_MOESM10_ESM.docx]

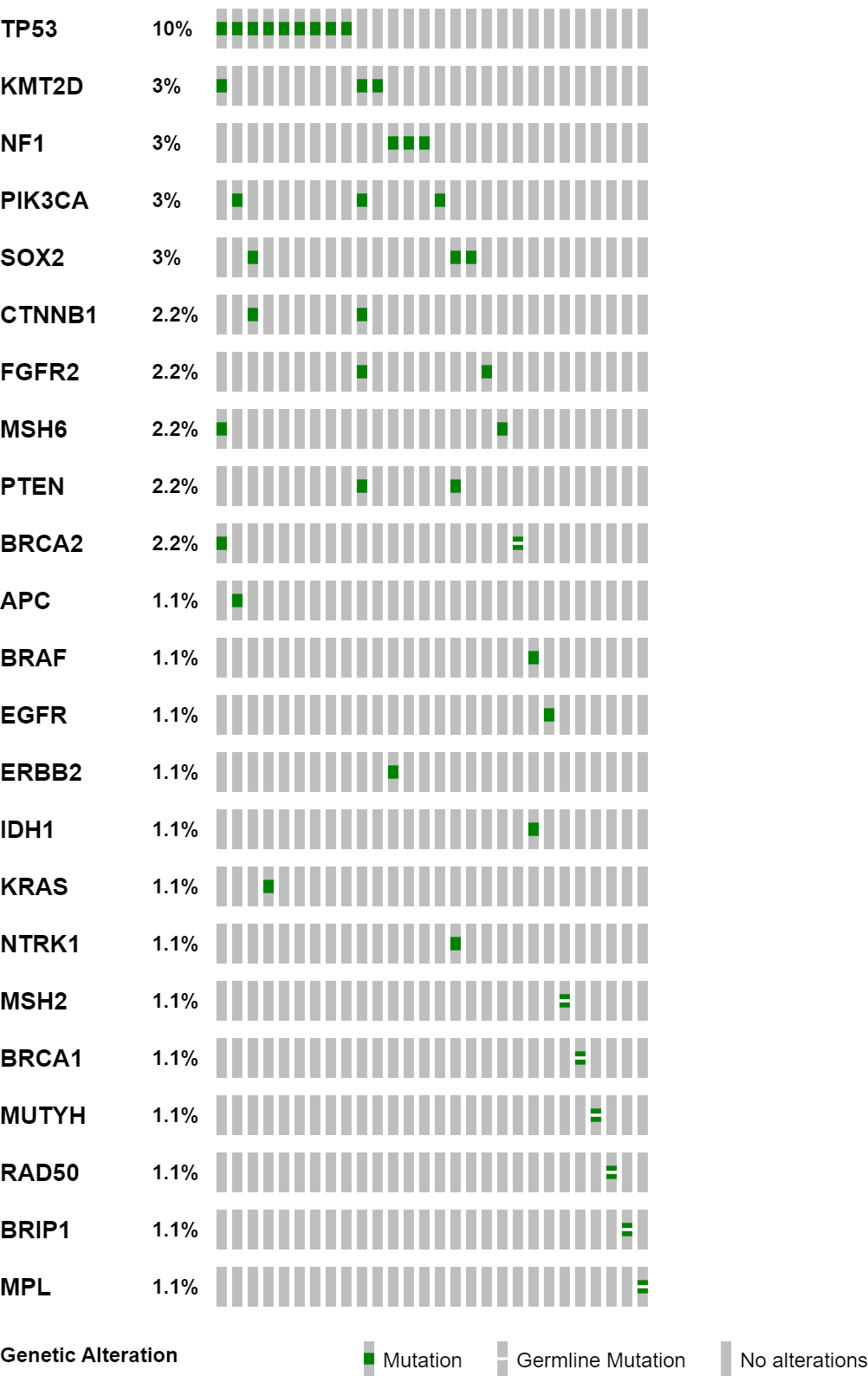

Supplement: Supplementary file 11 — Additional file 11: Figure S2. Oncoprint showing the distribution of genomic alterations in both somatic and germline genomes in cancer patients. [file 12885_2022_9525_MOESM11_ESM.docx]
